# The Ciliopathy Protein CC2D2A Associates with NINL and Functions in RAB8-MICAL3-Regulated Vesicle Trafficking

**DOI:** 10.1371/journal.pgen.1005575

**Published:** 2015-10-20

**Authors:** Ruxandra Bachmann-Gagescu, Margo Dona, Lisette Hetterschijt, Edith Tonnaer, Theo Peters, Erik de Vrieze, Dorus A. Mans, Sylvia E. C. van Beersum, Ian G. Phelps, Heleen H. Arts, Jan E. Keunen, Marius Ueffing, Ronald Roepman, Karsten Boldt, Dan Doherty, Cecilia B. Moens, Stephan C. F. Neuhauss, Hannie Kremer, Erwin van Wijk

**Affiliations:** 1 Institute for Molecular Life Sciences, University of Zurich, Zurich, Switzerland; 2 Institute of Medical Genetics, University of Zurich, Zurich, Switzerland; 3 Department of Otorhinolaryngology, Radboud University Medical Centre, Nijmegen, the Netherlands; 4 Radboud Institute for Molecular Life Sciences, Radboud University Nijmegen, the Netherlands; 5 Department of Human Genetics, Radboud University Medical Centre, Nijmegen, the Netherlands; 6 Department of Pediatrics, University of Washington, Seattle, Washington, United States of America; 7 Department of Biochemistry, University of Western Ontario, London, Ontario, Canada; 8 Department of Ophthalmology, Radboud University Medical Centre, Nijmegen, the Netherlands; 9 Division of Experimental Ophthalmology and Medical Proteome Center, Centre for Ophthalmology, Eberhard Karls University Tuebingen, Germany; 10 Fred Hutchinson Cancer Research Center, Seattle, Washington, United States of America; Washington University School of Medicine, UNITED STATES

## Abstract

Ciliopathies are a group of human disorders caused by dysfunction of primary cilia, ubiquitous microtubule-based organelles involved in transduction of extra-cellular signals to the cell. This function requires the concentration of receptors and channels in the ciliary membrane, which is achieved by complex trafficking mechanisms, in part controlled by the small GTPase RAB8, and by sorting at the transition zone located at the entrance of the ciliary compartment. Mutations in the transition zone gene *CC2D2A* cause the related Joubert and Meckel syndromes, two typical ciliopathies characterized by central nervous system malformations, and result in loss of ciliary localization of multiple proteins in various models. The precise mechanisms by which CC2D2A and other transition zone proteins control protein entrance into the cilium and how they are linked to vesicular trafficking of incoming cargo remain largely unknown. In this work, we identify the centrosomal protein NINL as a physical interaction partner of CC2D2A. NINL partially co-localizes with CC2D2A at the base of cilia and *ninl* knockdown in zebrafish leads to photoreceptor outer segment loss, mislocalization of opsins and vesicle accumulation, similar to *cc2d2a*-/- phenotypes. Moreover, partial *ninl* knockdown in *cc2d2a*-/- embryos enhances the retinal phenotype of the mutants, indicating a genetic interaction in vivo, for which an illustration is found in patients from a Joubert Syndrome cohort. Similar to zebrafish *cc2d2a* mutants, *ninl* morphants display altered Rab8a localization. Further exploration of the NINL-associated interactome identifies MICAL3, a protein known to interact with Rab8 and to play an important role in vesicle docking and fusion. Together, these data support a model where CC2D2A associates with NINL to provide a docking point for cilia-directed cargo vesicles, suggesting a mechanism by which transition zone proteins can control the protein content of the ciliary compartment.

## Author Summary

Ciliopathies are a group of disorders caused by dysfunction of primary cilia, ubiquitous organelles involved in signal transduction. Mutations in *CC2D2A* cause two ciliopathies, Joubert and Meckel syndromes, and result in loss of ciliary protein localization. The mechanism by which CC2D2A, located at the ciliary transition zone, controls ciliary protein composition and its link to vesicular trafficking of incoming cargo remain largely unknown. Here, we identify a series of physical interactions linking CC2D2A to vesicular trafficking controlled by the small GTPase RAB8, suggesting a new model, whereby CC2D2A provides a specific docking point for ciliary-bound vesicles at the entrance to the ciliary compartment. We first identify NINL as a physical and genetic interaction partner of CC2D2A, show that both proteins co-localize at the entrance to the cilium and demonstrate that absence of Ninl or Cc2d2a result in similar retinal phenotypes in zebrafish, including mislocalization of Rab8. We further identify MICAL3, a protein known to bind RAB8, as another NINL interaction partner, thus linking CC2D2A to RAB8A-controlled trafficking. Finally, we describe an individual with Joubert syndrome, in whom combined *CC2D2A* and *NINL* mutations result in an enhanced phenotype, illustrating the impact of the detected interaction on the disease.

## Introduction

Primary cilia are microtubule-based organelles protruding from the apical surface of most differentiated vertebrate cell types where they play a crucial role in transduction of extra-cellular signals to the cell [1]. Cilia achieve this function by concentrating and regulating receptors and channels that are required for sensing these signals in their membrane domain. Consequently, the ciliary membrane has a distinct composition from that of the adjacent plasma membrane, despite them being continuous with each other [2]. The tight regulation required to maintain the specificity of the ciliary membrane composition is achieved by complex trafficking and sorting mechanisms at the entry point to the ciliary compartment, as well as by a diffusion barrier present at the base of the cilium [3,4]. The transition zone, at the base of the ciliary axoneme, plays a crucial role in this sorting mechanism [5,6]. Indeed, dysfunction of proteins normally localized at the transition zone leads to both abnormal access to the ciliary compartment for proteins that should not localize there and loss of normal localization for ciliary proteins [5,7]. The actual mechanism, by which these transition zone proteins contribute to this sorting of ciliary proteins, remains however largely unknown.

Mutations in transition zone proteins in humans lead to several ciliopathies such as Joubert syndrome. Ciliopathies are a group of human disorders caused by dysfunction of primary cilia and characterized by overlapping genetics and phenotypes [8]. As cilia are present on most vertebrate cells, their dysfunction can manifest as a wide array of phenotypic features affecting most organs systems [9]. Retinal dystrophy is a common finding in ciliopathies given that retinal photoreceptor outer segments, which are the site of the phototransduction cascade, are highly specialized primary cilia [10]. Joubert syndrome (JBTS) (OMIM 213300) is a prototypical ciliopathy with a phenotypic spectrum that can encompass most of the typical ciliopathy phenotypes [11,12]. It is characterized by a specific hindbrain malformation termed the molar tooth sign (MTS), in addition to which affected individuals may have retinal dystrophy, tubulo-interstitial kidney disease, liver fibrosis, skeletal dysplasia and polydactyly [13–15]. To date, mutations in over 27 different genes have been reported as an underlying cause for JBTS [12,16–20]. Most of these genes encode proteins associated in multi-protein complexes localized at the transition zone of the primary cilium [7,21].

Mutations in *CC2D2A* (Coiled-coil and C2-domains containing protein 2A) are the second most common genetic cause for JBTS, accounting for almost 9% of affected individuals [12,22]. Moreover, mutations in *CC2D2A* can also result in the genetically related and more severe Meckel syndrome, which is a perinatal-lethal disorder characterized by encephalocele, polydactyly, cystic kidneys and liver fibrosis [23]. CC2D2A is part of one of the ciliary transition zone complexes with several other JBTS proteins [7,21]. Two *Cc2d2a* mouse mutants have been described, presenting with severe brain malformation (holoprosencephaly), microphthalmia, curved body axis and randomized left-right axis, all typical ciliopathy-associated phenotypes [7,24]. Interestingly, mouse embryonic fibroblasts from one of the reported *Cc2d2a* ^*-/-*^ mice appear to lack cilia entirely [24] whereas disruption of CC2D2A function in the other reported mutant does not compromise ciliogenesis in mouse embryonic fibroblasts [7]. Instead, the ciliary localization of several proteins (including ARL13B, Adenylyl Cyclase III, Smoothened and Polycystin2) is lost, suggesting that presence of CC2D2A at the transition zone is required for appropriate targeting of proteins to the ciliary compartment [7]. The zebrafish *cc2d2a* mutant *sentinel* demonstrates a curved body axis, pronephric cysts and a striking retinal phenotype with short and dysmorphic photoreceptor outer segments [25]. In addition, the photoreceptors of *cc2d2a* mutants also show mislocalization of opsins in the cell body and cytoplasmic accumulation of vesicles in the apical portion of the cells and around the connecting cilium (equivalent of the transition zone in photoreceptors), suggesting a defect in opsin trafficking. Opsins are the photosensitive pigment molecules concentrated at high levels in the outer segments and required for sensing the light signal. Trafficking of opsins from the cell body towards the ciliary compartment is (at least in part) controlled by the small GTPase Rab8, which coats rhodopsin-carrier vesicles allowing their docking and fusion at the ciliary base [26]. Expression of a dominant-negative form of Rab8a leads to accumulation of rhodopsin-containing vesicles in photoreceptors [27]. In addition, RAB8A is also involved in ciliary membrane biogenesis in other cell types and thus appears to play a general role in orchestrating trafficking towards the ciliary compartment [28,29]. The trafficking defect observed in *cc2d2a* ^-/-^ photoreceptors appears to be mediated by loss of normal Rab8 localization [25] but the precise mechanism by which loss of this transition zone protein affects the localization of Rab8 and the trafficking of ciliary-directed opsins remains unclear.

In the current work, we identify a chain of physical interactions linking CC2D2A to RAB8A through NINL and MICAL3. Using a zebrafish model, we demonstrate that loss of Ninl function leads to a similar retinal phenotype as loss of Cc2d2a, including short outer segments, mislocalization of opsins and accumulation of vesicles. Based on the physical and genetic interactions that we identify, we propose a model in which CC2D2A provides a docking point at the photoreceptor ciliary base, allowing RAB8A-positive vesicles to bind through a series of interactions involving CC2D2A-NINL-MICAL3-RAB8A.

## Results

### CC2D2A associates with NINL

**Fig 1:**
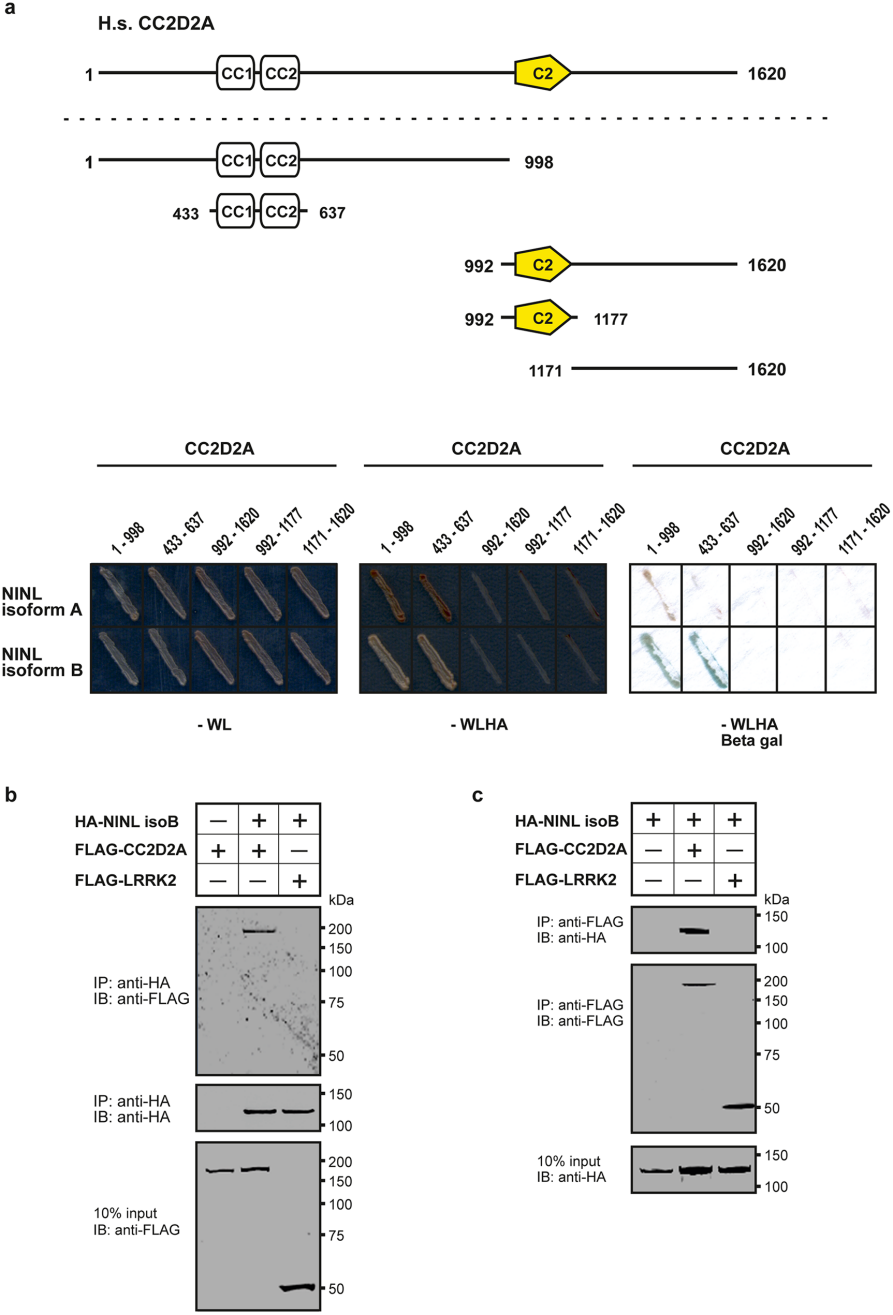
CC2D2A associates with NINL. (**a**) Yeast two-hybrid interaction assays were performed with different fragments of CC2D2A fused to the GAL4 DNA binding domain (BD) and full length NINL isoform A and B, fused to the GAL4 activation domain (AD). Activation of the reporter genes, which indicates a physical interaction, was dependent on coiled-coil (CC) domains 1 and 2 of CC2D2A and either NINL isoform A or B. (**b**) The top panel of the immunoblot (IB) shows that FLAG-tagged CC2D2A, but not the FLAG-tagged LRRK2 that was included as a negative control, was co-precipitated with HA-tagged NINL isoform B using a rat monoclonal antibody directed against the HA-epitope. Protein input is shown in the lower panel; anti-HA precipitates are shown in the middle panel. (**c**) In a reciprocal experiment, HA-tagged NINL^isoB^ was co-precipitated with FLAG-tagged CC2D2A, but not with FLAG-tagged LRRK2. Protein input is shown in the lower panel; anti-FLAG precipitates are shown in the middle panel.

In order to shed light on the function of CC2D2A we performed a dedicated ciliary yeast two-hybrid assay with different fragments of CC2D2A against a panel of 164 proteins, containing most of the ciliopathy-associated proteins [30]. A direct binary interaction between CC2D2A and both isoforms (A and B) of the centrosome- and basal body-associated protein NINL (Ninein-like protein) was identified (Fig 1a). NINL isoforms A and B are distinguished by the fact that isoform B is 349 amino acids shorter due to skipping of the large exon 17 (S1a Fig). Both isoforms share predicted EF-hand domains in the N-terminal region as well as coiled-coil domains in the more C-terminal portion. Both isoforms display similar broad expression patterns, with the strongest expression patterns in cochlea, brain, testis, kidney and retina [31].

By generating deletion constructs for CC2D2A and subsequent evaluation of the interaction with NINL, we could pinpoint the interaction to the two predicted coiled-coil domains (433-637aa) present in CC2D2A (Fig 1a). Since CC2D2A and NINL isoform B demonstrated the strongest interaction (Fig 1a), we focused on NINL isoform B (NINL^isoB^) for confirmation and further investigation of this interaction. Co-immunoprecipitation assays performed using full-length tagged-constructs for NINL^isoB^ and CC2D2A, showed co-precipitation of the two proteins. FLAG-tagged LRRK2 that was used as a negative control did not co-precipitate with HA-tagged NINL^isoB^, which confirmed the specificity of the interaction between NINL^isoB^ and CC2D2A in this assay (Fig 1b). A reciprocal co-immunoprecipitation experiment confirmed the interaction between NINL^isoB^ and CC2D2A (Fig 1c).

### CC2D2A and NINL co-localize at the base of cilia independently of each other

**Fig 2:**
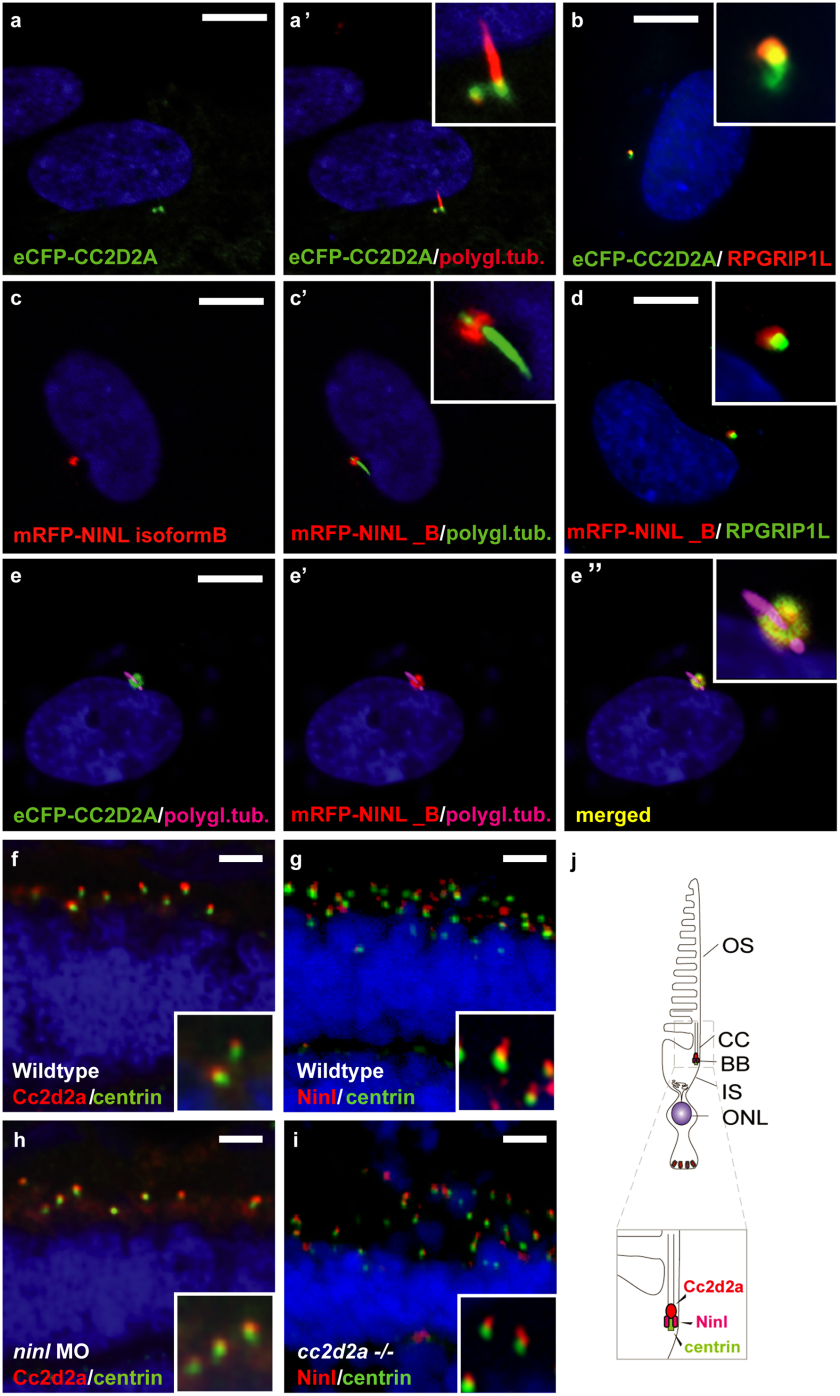
CC2D2A and NINL co-localize at the ciliary base in hTERT-RPE1 cells and in zebrafish retina. (**a, a’** and inset) When expressed alone, eCFP-tagged CC2D2A (green signal) localizes to the ciliary base (basal body, accessory centriole). The cilium is marked by anti-polyglutamylated tubulin (red signal, **a’** and inset). eCFP-tagged CC2D2A (green signal; **b**) also (partly) localizes to the ciliary transition zone, which was visualized using anti-RPGRIP1L as a marker (red signal; **b**). (**c, c’** and inset) mRFP-tagged NINL isoform B was localized at the ciliary base (cilium in green, **c’** and inset). (**d** and inset) mRFP-tagged NINL isoform B (red signal) localizes adjacent to the ciliary transition zone (anti-RPGRIP1L; green signal). (**e-e”** and inset) Co-expression of mRFP-tagged NINL isoform B (red signal) and eCFP-tagged CC2D2A showed co-localization of both proteins around the ciliary base (yellow signal). (**f**) In wild-type larval zebrafish retina (4 dpf), Cc2d2a marked by anti-Cc2d2a antibodies (red signal) is localized apically to the photoreceptor basal body (marked by anti-centrin antibodies, green signal). (**g**) Ninl, stained with anti-Ninl antibodies, (red signal) is localized at the zebrafish photoreceptor ciliary base, partially overlapping with and apical to the green centrin signal. (**h**) Cc2d2a localization is unaffected by *ninl* knockdown and (**i**) Ninl localization is normal in *cc2d2a* ^**-/-**^ larvae. (**j**) Schematic representation of the localization of Ninl and Cc2d2a in zebrafish photoreceptor cells. (**f-i**) are immunostainings on cryosections from 4 dpf larvae. Nuclei were stained with DAPI (blue signal) in all panels. Scale bars are 10 μm in a-e, and 4 μm in f-i.

To further validate the interaction between CC2D2A and NINL^isoB^ in ciliated mammalian cells, we transfected hTERT-RPE1 cells (human telomerase reverse transcriptase retinal pigment epithelium cells) with expression-constructs of wild-type mRFP-tagged NINL^isoB^, eCFP-tagged CC2D2A or a combination of both. When expressed alone, eCFP-tagged CC2D2A localizes to the ciliary base (basal body, accessory centriole) and also (partly) to the ciliary transition zone, which was visualized using anti-RPGRIP1L as a marker (Fig 2a and 2b). mRFP-tagged NINL isoform B was localized at the ciliary base adjacent to the ciliary transition zone (Fig 2c and 2d). When co-expressed, NINL^isoB^ and CC2D2A co-localized at the base of the primary cilium (Fig 2e-e”).

To investigate the localization and function of endogenous Ninl, we turned to the zebrafish model. The zebrafish genome harbors a single *ninl* orthologue (Genbank NP_001268727) that has 45% similarity with human *NINL*. Conserved domains include the predicted EF-hand domains and multiple coiled-coil domains. Cloning of zebrafish *ninl* from whole embryo mRNA at 5dpf revealed that all identified zebrafish transcripts lack the large exon 17 which is present only in human NINL isoform A but not in isoform B (S1A Fig). Therefore, zebrafish *ninl* is most similar to the shorter human NINL isoform B. RNA *in situ* hybridization with two different antisense probes derived from the 5’-end and the 3’-end of the zebrafish *ninl* transcript revealed broad expression at 14–18 somites in neural tube, inner ear, developing eye and pronephros. Expression persists in the retina at least up to 6 dpf (days post fertilization; last developmental stage assessed) (S1B Fig). Antibody staining showed punctate localization of endogenous Ninl in zebrafish retina at the base of the cilium in 4dpf larvae (Fig 2g). While co-staining with Cc2d2a antibodies was not possible due to different fixation conditions, co-staining of serial sections with anti-centrin and anti-Ninl or anti-Cc2d2a antibodies respectively revealed that both endogenous proteins partially co-localize at the base of the photoreceptor cilium of 4 dpf old zebrafish larvae (Fig 2f and 2g). We observed that Cc2d2a localizes slightly more apically with respect to Centrin than Ninl, consistent with Cc2d2a localization at the connecting cilium, while Ninl localization is overlapping more broadly with the basal body (Fig 2f and 2g, schematized in j).

In order to determine whether Cc2d2a localization is dependent on the presence of Ninl, we performed morpholino-induced knockdown studies in zebrafish. Injection of 2 ng/nl of *ninl* translation-blocking morpholino (atgMO) led to efficient knockdown of Ninl, as demonstrated by substantially decreased antibody staining in cryosections through morphant retina (S2a and S2b” Fig). On Western blots of whole 5dpf larval extracts, a single strong band of 80 kDa is present in wild-type fish (S2d Fig), which is consistent with results from immunoprecipitation from retinal bovine extracts with a previously published antibody against human *NINL* (S2e Fig [31]). This band is strongly reduced in *ninl* atgMO injected larvae (S2d Fig), supporting the specificity of the morpholino and of the antibody.

Ninl knockdown led to typical ciliopathy-associated phenotypes, including curved body shape, enlarged brain ventricle and pronephric cysts (S3a–S3g Fig). The specificity of the observed phenotype was confirmed by rescue experiments, in which co-injection of 2 ng/nl *ninl* MO with capped MO-resistant human *NINL-*mRNA reduced the prevalence of the curved body phenotype in a dose-dependent manner (curved body shape in 71% of *ninl* atgMO injected larvae (n = 207) versus 36% in *ninl* atgMO + *ninl* mRNA injected larvae (n = 203), data pooled from 2 biological replicates, *P*<0.0001, two-tailed Fisher’s exact test; S4a–S4d Fig). Finally, the specificity of the observed phenotypes was further confirmed by a second morpholino against *ninl* targeting the splice site at the intron14/exon15 junction and thus causing aberrant splicing with premature truncation (S5c Fig). This splice morpholino led to similar phenotypes as the atgMO, including ventriculomegaly and abnormal photoreceptor outer segments (S5a and S5b Fig). The body curvature phenotype was absent in the splice morphants, which may be explained either by rescue of this early phenotype by maternal *ninl* mRNA, which remains unaffected by splice morpholinos (as seen in some ciliopathy zebrafish mutants such as *talpid3* where only the maternal zygotic mutants have a curved body shape [32]), or by less efficient gene knockdown with this morpholino, as normal transcript persists in addition to the aberrant transcript (S5c Fig). Indeed, using the anti-NINL antibody, we observed a milder decrease of Ninl protein on Western blots and on immuno-histochemistry of retinal cryosections at 5dpf for the *ninl* ex15 spMO as compared to the atgMO (S2c and S2d Fig).

Localization of Cc2d2a at the connecting cilium, shown by anti-Cc2d2a immunostaining, was unaffected by Ninl knockdown (Fig 2h). Conversely, immunostainings using anti-Ninl antibodies revealed no clear mislocalization of Ninl in the retina of *cc2d2a* ^-/-^ larvae (Fig 2i). Taken together, these data indicate that Cc2d2a and Ninl co-localize at the ciliary base independently of each other.

### NINL knockdown in zebrafish leads to outer segment loss, opsin mislocalization and vesicle accumulation

**Fig 3:**
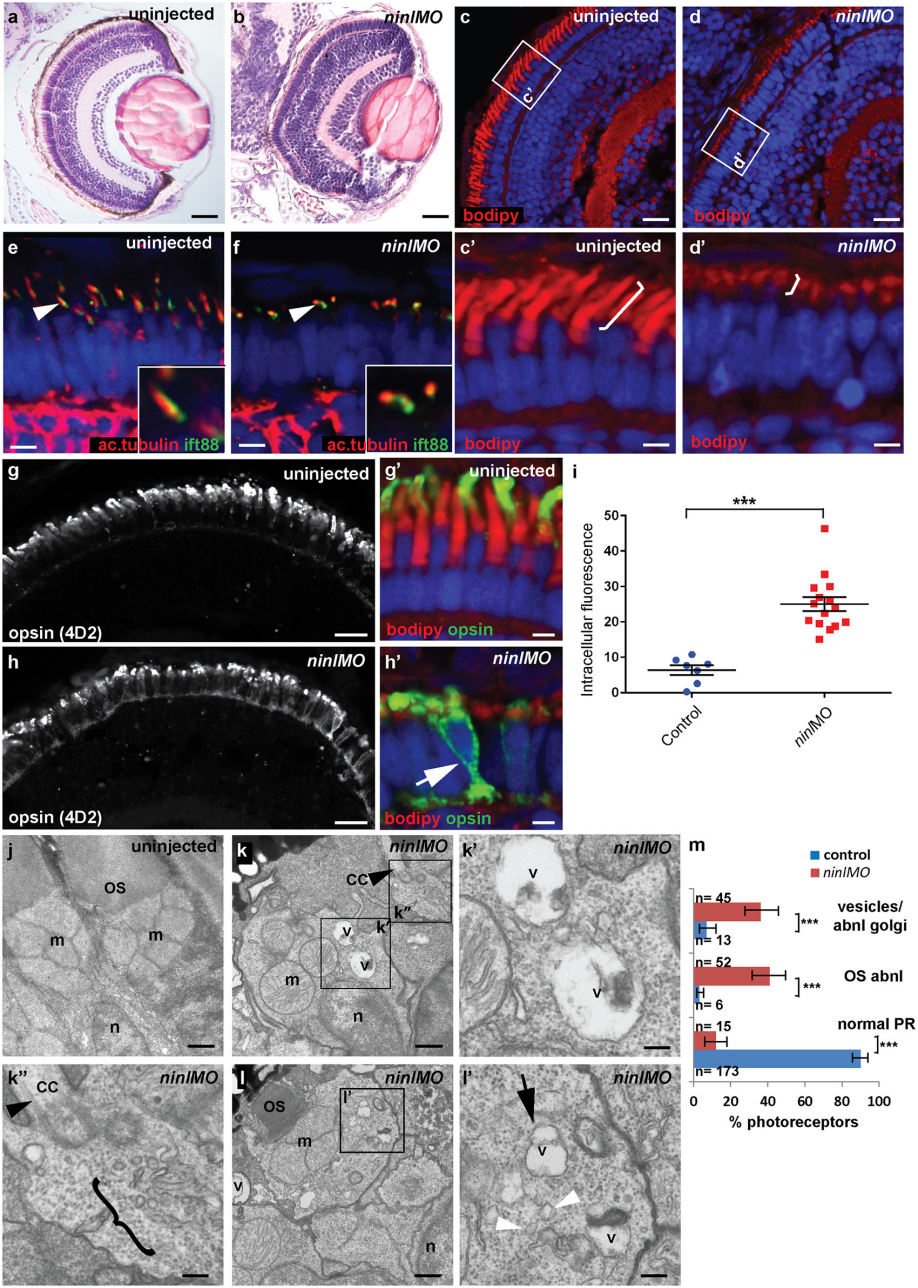
Zebrafish *ninl* knockdown causes loss of axonemes and outer segments, opsin mislocalization and vesicle/vacuole accumulation. (**a-b**) Paraffin sections stained with Hematoxylin/Eosin of control (**a**) and *ninl* knockdown larvae (**b**) demonstrating shortened outer segments and grossly preserved retinal lamination in the morphants. (**c-d’**) Bodipy-stained cryosections highlight the shortened (brackets **c’-d’**) and dysmorphic outer segments of ninl knockdown larvae (**d** and **d’**) compared to the long cone- or rod-shaped outer segments of controls (**c** and **c’**). (**e-f**) Axonemes and connecting cilia marked with anti-acetylated alpha-tubulin and anti-Ift88 antibodies are severely shortened and reduced in numbers in *ninl* knockdown larvae (arrowhead in f). (**g-h’**) Immunofluorescence with anti-opsin antibody 4D2 demonstrates mislocalization of opsins within the cell body in *ninl* knockdown larvae (arrow in **h’**) compared to controls (**g**) where opsins are restricted to the outer segment. (**i**) Quantification of the intracellular opsin accumulation in *ninl* morphant photoreceptors compared to control: each single datapoint in the scatter graph displays the averaged mean grey value from one larva. The mean value and the Standard Error of the Mean (SEM) are displayed as bars. The difference is statistically significant (*** = p<0.0001, Student’s *t*-test). (**j-l’**) Transmission electron microscopy of control (**j**) and *ninl* knockdown larvae (**k-l’**) demonstrates absent or shortened and dysmorphic outer segments (OS) and accumulation of large vacuoles (v, arrow in **l’**) and smaller vesicular structures (bracket in **k”** and white arrowheads in **l’**) in morphants. Black arrowheads point to the connecting cilium in k and k”. k’ and k” are the boxed areas in k and l’ is the boxed area in l. (**m**) Quantification of the % of photoreceptors displaying these phenotypes. Absolute numbers of photoreceptors are also indicated. Error bars indicate 95% Confidence Intervals. The differences between morphant (red bars) and controls (blue bars) are statistically significant (*** = p<0.0001, Fisher’s exact test). Larvae in all panels are 4 dpf old. Scale bars are 30 μm in **a-b**, 15 μm in **c-d and g-h**, 3 μm in **c’-d’ and g’-h’**, 4 μm in **e-f**, 0.5 μm in **j-k and l** and 150nm in **k’-k”** and **l’**. OS outer segment, CC connecting cilium, m mitochondria, n nucleus, v vacuole.

Since *cc2d2a* ^*-/-*^ zebrafish have prominent retinal abnormalities [25], we focused our phenotypic analysis on the retina of *ninl* morphants. Retinal lamination was unaffected in *ninl* morphants (Fig 3a and 3b). In contrast, photoreceptors demonstrated shortened axonemes and abnormal outer segments, as seen on retinal cryosections at 4 dpf stained with boron-dipyrromethene (bodipy) to mark the outer segment membrane disks (Fig 3c and 3d’) and anti-acetylated alpha-tubulin and anti-Ift88 antibodies to mark the axoneme (Fig 3e and 3f). Measurement of outer segment (OS) length of early 4dpf larvae, performed in a blinded manner as to injection status, revealed a significant shortening (mean OS length 1.6 +/- 0.26 μm in *ninl* atgMO morphants compared to 3.9 +/- 0.32 μm in wild-type, *P*<0.0001, unpaired Student’s t-test, n>10 larvae from each group in each of 2 biological replicates; S4e Fig). This retinal phenotype was observed with both the *ninl* translation-blocking and the splice-blocking morpholinos (S5b’ Fig). Little to no photoreceptor cell death was observed with TUNEL assay on cryosections of 4dpf morphant larvae (S3i and S3j Fig) compared to *ift88*-/- retinas that are known to display prominent photoreceptor cell death at the same stage (and were thus used as positive controls; S3k Fig). In general, minimal cell death was observed at 4dpf throughout the embryo, including in brain of larvae with overt ventriculomegaly (S3l Fig). Co-injection of 150pg of capped human *NINL* mRNA with 2ng/nl *ninl* atgMO restored normal outer segment length (mean OS length in rescued larvae 3.8 +/- 0.25 μm, *P*<0.0001, unpaired Student’s *t*-test, n = 10 larvae; S4a’–S4c’ and S4e Fig).

Immuno-staining with anti-opsin antibodies (4D2 antibody) demonstrated significant accumulation of opsins in the inner segment and throughout the cell body of *ninl*-depleted photoreceptors (Fig 3g–3i; mean intracellular fluorescence was significantly increased in *ninl* morphants compared to controls, *P*<0.0001, unpaired Student’s *t*-test, n = 15 morphant larvae and 7 control larvae, 2 replicate experiments). At the ultra-structural level, two types of abnormal membrane-bound structures were observed by transmission electron microscopy in *ninl* morphants: large vacuole-like structures were present in the cell body and small vesicular structures accumulated around the Golgi complex and below the connecting cilium (Fig 3j–3m; vacuolar and/or vesicular structures were present in 45/112 photoreceptors from 6 morphant eyes compared to 13/192 photoreceptors from 4 uninjected and 4 Control Oligo injected eyes; *P*<0.0001, Fisher’s exact test). These phenotypes are partially reminiscent of those observed in *cc2d2a* ^-/-^ embryos [25], supporting a common or coordinated function for Cc2d2a and Ninl in the process of vesicular trafficking towards the ciliary compartment.

### *Ninl* genetically interacts with *cc2d2a* and may act as a genetic modifier for *CC2D2A*-associated Joubert Syndrome

**Fig 4:**
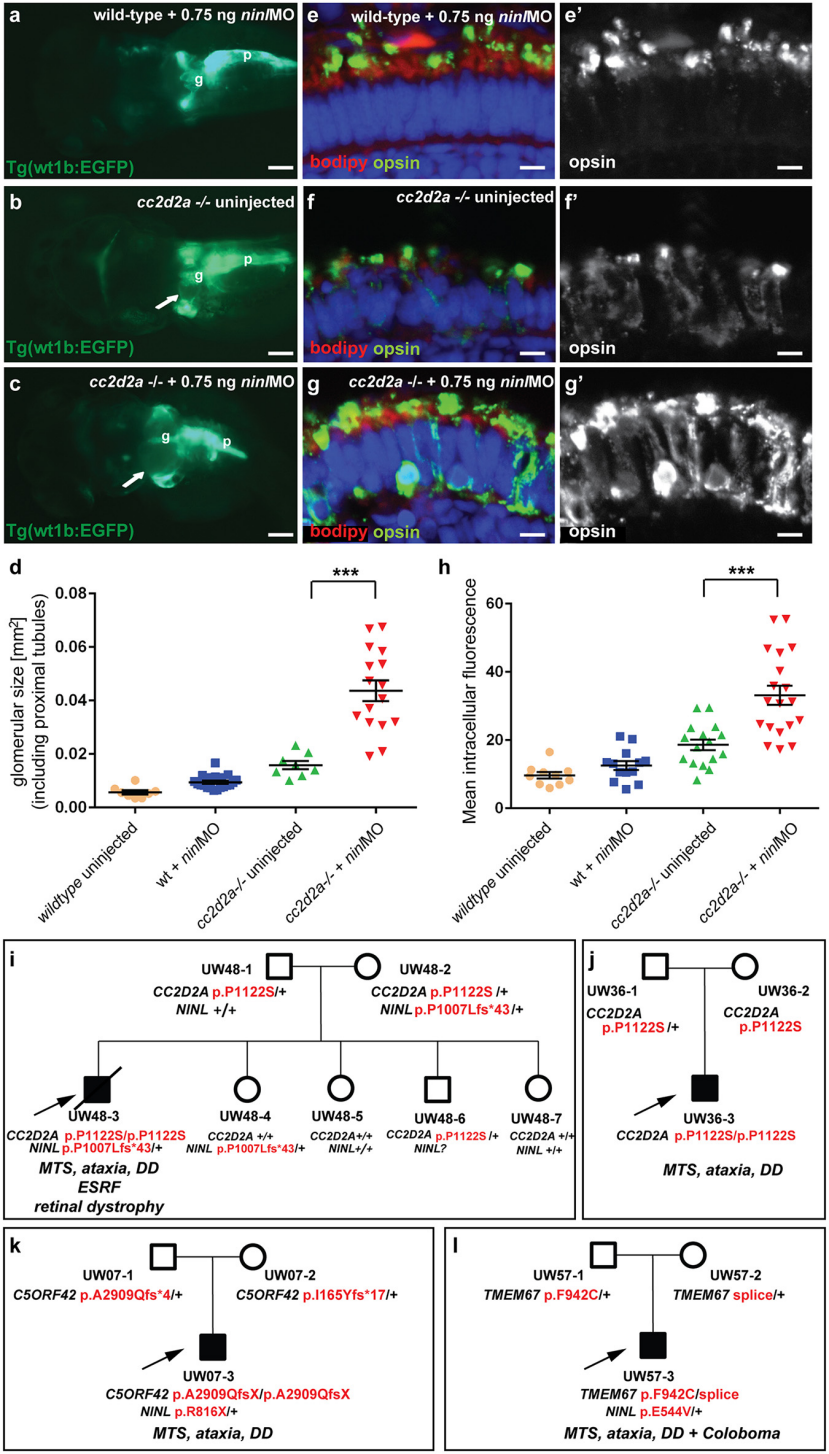
Genetic interaction between *ninl* and *cc2d2a*. (**a-d**) Partial *ninl* knockdown enhances the cystic kidney phenotype of *cc2d2a* mutants. (**a-c**) Glomerulus and proximal pronephric tubules highlighted in the transgenic line Tg(wt1b-EGFP). (**a**) Injection of a low dose of *ninl* atgMO (0.75 ng/nl) causes no cysts in wild-type larvae. (**b**) *cc2d2a*-/- larvae display small dilatations of the proximal tubules (arrow) in ~40% of cases. (**c**) Injection of this low dose of *ninl* atgMO in the *cc2d2a*-/- background leads to large dilatations of the proximal tubules and glomerular space (arrow) in 89% of mutants. ***g*** glomerulus, ***p*** pancreas. (**d**) Quantification of the glomerular + proximal tubular area displayed as a scatter plot, demonstrating a significant increase in proximal pronephric area in *cc2d2a*-/- larvae injected with low-dose *ninl* atgMO. The bars represent the mean and standard error of the mean (SEM) for each treatment group and each datapoint is an individual fish. (**e-g’**) Immunohistochemistry with anti-opsin antibody (4D2, green) on retinal cryosections of 4dpf *cc2d2a*-/- uninjected larvae (**f-f”**) and *cc2d2a*-/- larvae injected with subphenotypic doses of *ninl* MO (**g’g”’**), that cause no mislocalization in wild-type fish (**e-e’**), demonstrates that partial *ninl* knockdown increases the mislocalization of opsins (**e’-g’**). (**h**) Quantification of the mean intracellular fluorescence displayed as a scatter plot shows significant increase in intracellular fluorescence in *cc2d2a*-/- larvae injected with low dose of *ninl* atgMO. The bars represent the mean and standard error of the mean (SEM) for each treatment group and each datapoint represents the mean intracellular fluorescence from 10 photoreceptors in one individual fish. Cell membrane and outer segments are stained with bodipy (red in **e-**g). Nuclei are counterstained with DAPI. Scale bars are 100 μm in (a-c) and 4 μm in (e-g’). (**i**) Pedigree of a consanguineous family with one affected boy (UW48-3) and 4 unaffected siblings. UW48-3 carried a homozygous missense *CC2D2A* mutation as well as a frameshift mutation in *NINL* leading to premature truncation. (**j**) Pedigree of a family where the affected individual (UW36-3) carries the same homozygous *CC2D2A* mutation as in (**i**) but no additional rare deleterious variants. (**k**) Pedigree of a family where the affected individual (UW07-3) carries compound heterozygous *C5ORF42* frameshift mutations and a nonsense mutation in *NINL*. (**l**) Pedigree of a family where the affected individual (UW57-3) carries compound heterozygous *TMEM67* mutations and a missense *NINL* mutation. The phenotype of the affected individuals is detailed in *italic* on each pedigree under the corresponding mutations. *MTS* Molar Tooth Sign, *DD* Developmental Delay, *ESRF* End-Stage Renal Failure.

To further delineate the relationship between *cc2d2a* and *ninl*, we tested whether a synergistic effect was detectable between the two genes by using partial *ninl* knockdown in the *cc2d2a* mutant background. We observed that injection of a sub-phenotypic dose of *ninl* MO (0.75 ng/nl), which causes no discernible phenotype in wild-type larvae, significantly increased the penetrance and severity of pronephric cysts in *cc2d2a* mutants: 89% of *ninlMO-cc2d2a* ^*-/-*^ zebrafish developed cysts compared to 40% of uninjected *cc2d2a* ^-/-^ larvae (p<0.0001, Fisher’s exact test) and the size of these cysts was significantly increased (as measured by the area of the dilated glomerulus and proximal tubules: 0.044 +/-0.004 mm^2^ for *cc2d2a*-/- + *ninl*MO (n = 16) as compared to 0.016 +/-0.002 mm^2^ for uninjected *cc2d2a*-/- (n = 8, *P*<0.0001, unpaired Student’s *t-*test) (Fig 4a–4d). Importantly, the *cc2d2a*+/- and *cc2d2a*+/+ siblings from the same injection clutch did not develop pronephric cysts at these sub-phenotypic *ninl* MO doses (Fig 4a and 4d). In the retina, the opsin mislocalization phenotype in *cc2d2a* ^-/-^ larvae (4 dpf) was enhanced by the addition of the same sub-phenotypic dose of *ninl*MO (Fig 4e–4h; *P*<0.0001, Student’s *t*-test, n = 19 *cc2d2a*-/- + *ninl*MO and n = 16 *cc2d2a*-/- uninjected, 2 replicates). These findings support a genetic interaction between *cc2d2a* and *ninl* and suggest that *NINL* could be a genetic modifier for *CC2D2A*-caused disorders or even contribute to the genetic spectrum underlying Joubert/Meckel syndrome. Following this rationale, we sequenced *NINL* in a cohort of 346 individuals with Joubert syndrome (from 291 families) using a molecular inversion probes (MIPs) capture method followed by next-generation sequencing [33] but did not identify any individuals carrying bi-allelic rare deleterious *NINL* variants. We did however find 3 individuals with heterozygous *NINL* mutations predicted to be deleterious. Individual UW48-3 carried the homozygous missense *CC2D2A* mutation c. 3364C>T (p.P1122S), previously shown to be causal for Joubert syndrome, and a heterozygous *NINL* frameshift mutation leading to a stop codon after 43 amino acids (c.3020delC, p.P1007Lfs*43) (Fig 4i) (and no other rare deleterious variant in any of the known JS genes). Phenotypically, this subject had a severe form of JBTS with retinal dystrophy, hearing loss, ventriculomegaly in addition to the MTS and renal failure leading to death at age 7 years. In comparison, subject UW 36–3 carried the same homozygous *CC2D2A* c.3364C>T (p.P1122S) mutation but no additional *NINL* variants (or rare deleterious variants in other JBTS genes) and presented with the “pure JBTS” phenotype, consisting only of the MTS with associated ataxia, developmental delay and respiratory rhythm disturbance (Fig 4j). Subject UW07-3 carried a heterozygous *NINL* nonsense mutation (c.2446 G>A, p.R816X) in addition to causal, compound heterozygous *C5ORF42* frameshift mutations (c.8726delG; p.A2909Qfs*4 and c.493delA, p.I165Yfs*17). This subject presented a classical Joubert phenotype without extra-neuronal manifestations, suggesting that the additional *NINL* frameshift had no effect on the clinical manifestations (Fig 4k). Finally, subject UW57-3 carried a heterozygous *NINL* missense mutation (c.1631A>T, p.E544V), predicted to be deleterious by Polyphen2, along with bi-allelic causal *TMEM67* mutations (c.2825T>G, p.F942C and c.978+3 A>G). This individual had Joubert syndrome with coloboma but no retinal, renal or hepatic involvement (Fig 4l). Given the known association between *TMEM67* mutations and coloboma [12,34], this additional feature is most likely explained by the causal gene mutations, while the additional *NINL* variant appears to have no obvious effect on the phenotype in individual UW57-3. While it remains possible that additional sequence variants in non-JBTS genes also contributed to the enhanced phenotype in individual UW48-3 and while our findings from a large human cohort remain of anecdotal nature given the rarity of this highly heterogeneous genetic disorder, taken together with the zebrafish experiments, they suggest that *NINL* may act as a genetic modifier specifically for *CC2D2A*-caused Joubert syndrome.

### Ninl is required for correct Rab8a localization

**Fig 5:**
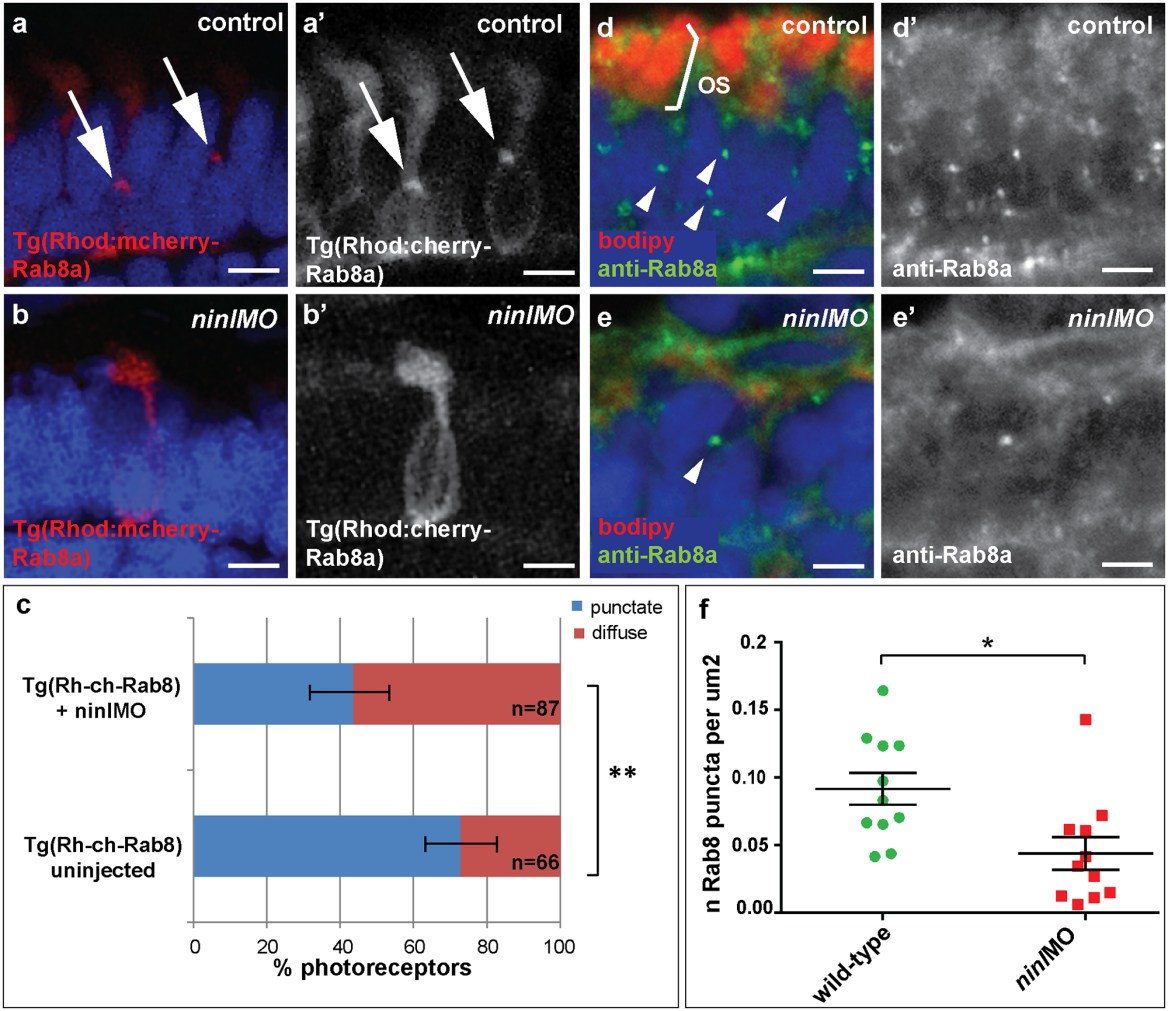
Ninl is required for correct Rab8A localization. (**a-a’**) Expression of a rhodopsin-promoter driven cherry-tagged Rab8a in wild-type photoreceptors is mostly concentrated in one or several puncta (arrows **a-a’**) whereas it is diffuse in the majority of *ninl* morphant photoreceptors (**b-b’**). (**c**) Proportion of Rab8a-cherry expressing photoreceptors with punctate expression versus diffuse expression (bars represent 95% confidence interval; ** *P*<0.001, *Fisher’s* exact test). (**d-d’**) Endogenous Rab8a localization as seen by immunohistochemistry using an anti-Rab8a antibody (green) displays similar puncta (arrowheads) in wild-type photoreceptors, while the number of puncta is decreased in *ninl* morphant photoreceptors (**e-e’**). (**f**) Quantification of the number of Rab8a puncta displayed in the form of a scatter plot indicating that significantly fewer endogenous Rab8 puncta per μm^2^ are present in *ninl* morphants compared to uninjected controls (*P = 0.*01*, unpaired Student’s *t-*test; bars represent standard error of the mean). Scoring was performed blinded as to injection status for (**c**) and (**f**). Outer segments are counterstained with bodipy in (**d-e**). Nuclei are counterstained with DAPI. All images are cryosections of 4 dpf larvae. Scale bars are 4 μm in all panels.

Previous work on the *cc2d2a* ^*uw38*^ zebrafish mutant demonstrated that loss of Cc2d2a leads to abnormal Rab8a localization in retinal photoreceptors [25]. Given the opsin mislocalization and vesicle accumulation phenotypes observed in *ninl* morphants, the known role of Rab8a in opsin trafficking [27,35,36] and the interaction with *cc2d2a* demonstrated here, we next determined whether loss of Ninl function also had an effect on Rab8a localization. For this purpose, we used a transgenic construct that drives expression of mCherry-tagged Rab8a in wild-type zebrafish photoreceptors in a punctate manner [25]. When expressed in *ninl* morphants (atgMO), mCherry-tagged Rab8a localized in significantly fewer puncta than when expressed in controls (42% of expressing photoreceptors displayed Rab8 puncta in *ninl* morphants (n = 38/87 from 14 larvae) compared to 73% in uninjected controls (n = 48/66 from 13 larvae), *p* = 0.0005, two-tailed Fisher’s exact test; Fig 5a–5c). Instead, expression of the transgene was mostly diffuse throughout the photoreceptor cell body of *ninl* morphants. A similar result was obtained using an anti-Rab8a antibody that recognizes endogenous small Rab8a puncta, which are found throughout the cell body, concentrated at the synapse and in the inner and outer segments in controls (Fig 5d-d’). In *ninl*-knockdown larvae, the number of endogenous Rab8a puncta was significantly reduced (Fig 5e-e’ and quantification in f: the average number of puncta per μm^2^ was reduced to 0.04 +/- 0.01 (or 1 puncta per 25 μm^2^) in *ninl* morphants as compared to 0.09 +/- 0.01 (or 1 puncta per 11 μm^2^) in uninjected wild-type, *P* = 0.01, unpaired Student’s t-test), supporting a role for Ninl in Rab8 localization.

### MICAL3 associates with NINL and is mislocalized in NINL and CC2D2A-depleted cells

**Fig 6:**
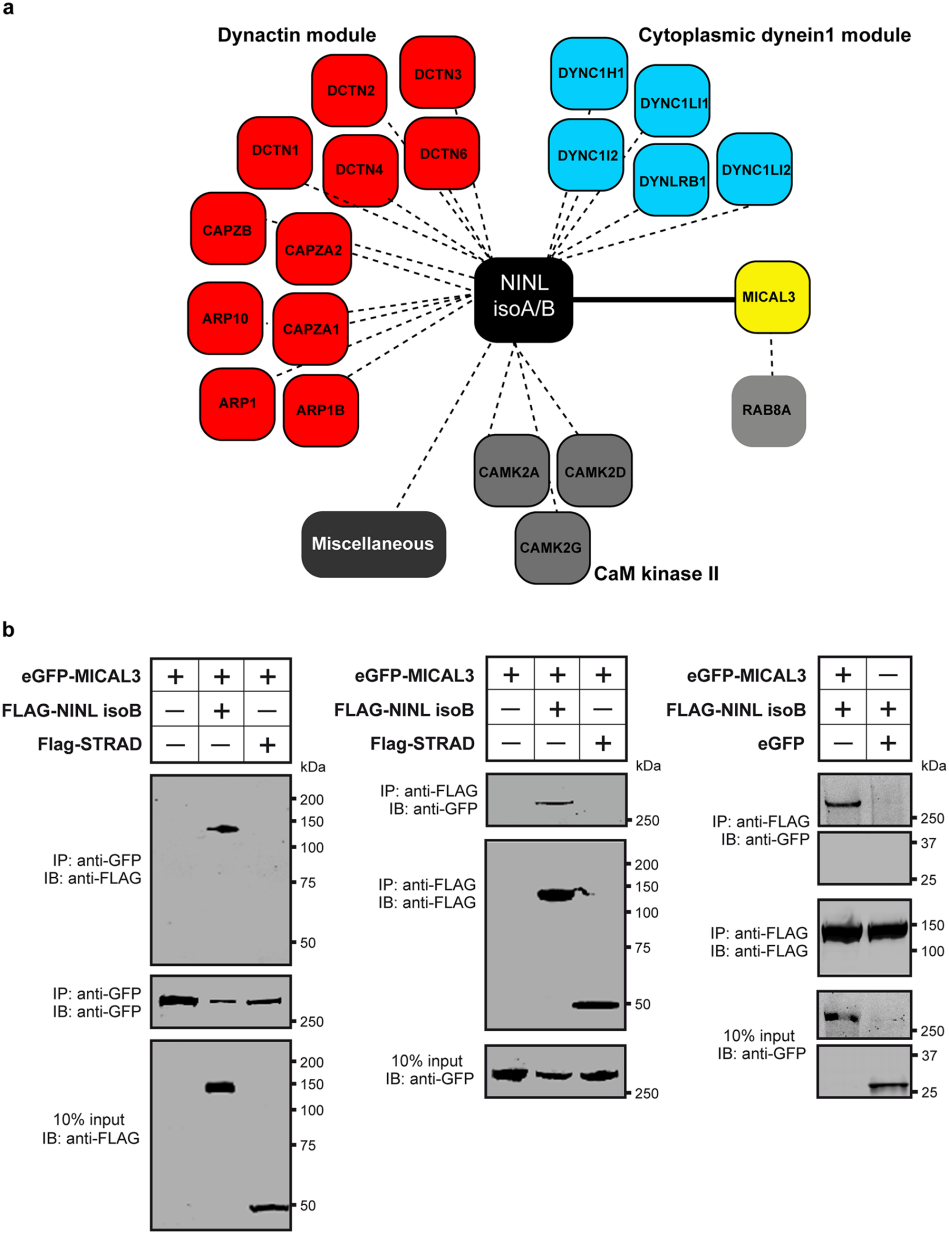
NINL interactome screen identifies MICAL3. (**a**) Strep-SILAC and TAP (tandem affinity purification) experiments show that NINL interacts specifically with MICAL3 (Yellow). The solid line between NINL and MICAL3 symbolizes a direct interaction, whereas the dashed lines indicate interactions determined by IP. (**b**) Co-immunoprecipitation of eGFP-MICAL3 with FLAG-NINL^isoB^, but not with FLAG-STRAD. The immunoblot (IB) in the top panel shows that eGFP-tagged MICAL3 co-immunoprecipitated with FLAG -tagged NINL (lane 2), whereas FLAG-tagged STRAD used as a negative control (lane 3) did not. The anti-GFP immunoprecipitates are shown in the middle panel; protein input is shown in the bottom panel. Reciprocal IP experiments using anti-FLAG antibodies confirmed the co-immunoprecipitation of eGFP-tagged MICAL3 with FLAG-tagged NINL^isoB^ (lane 2) and not with STRAD (lane 3) shown in the top panel. The anti-FLAG immunoprecipitations are shown in the middle panel; protein input is shown in the bottom panel. A co-immunoprecipitation experiment using untagged eGFP as a negative control (right panel) showed that eGFP-tagged MICAL3 immunoprecipitates with FLAG-tagged NINL^isoB^ but not with untagged eGFP.

**Fig 7:**
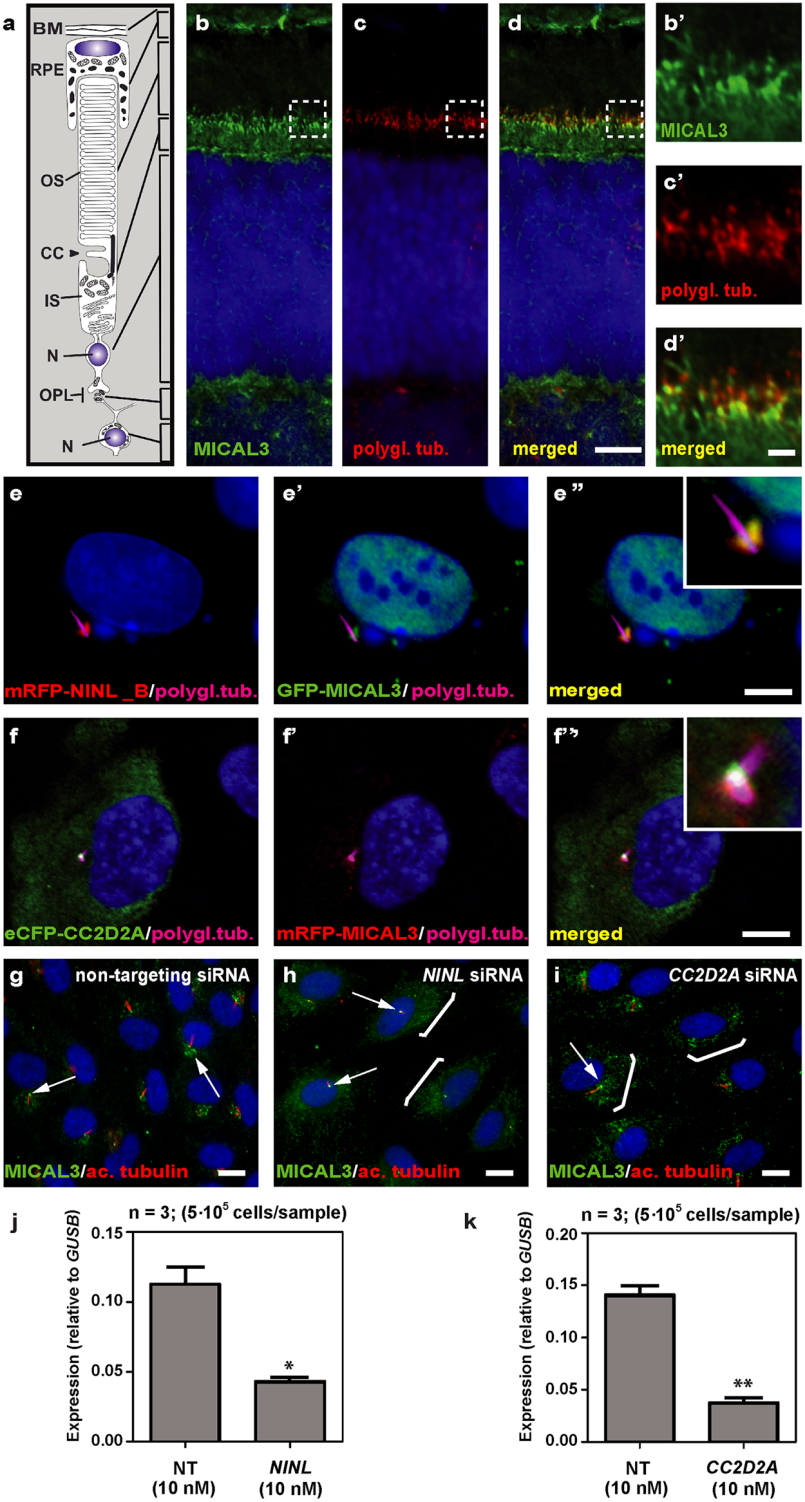
NINL and CC2D2A co-localize with MICAL3 and are required for correct MICAL3 localization. (**a**) Schematic of a photoreceptor for orientation. (**b-d’**) Co-localization of endogenous MICAL3 (green signal; **b**) and polyglutamylated tubulin (red signal; **c**) in rat retina (P20) by co-immunostaining radial cryo-sections. The yellow signal in the merged image (**d’**) indicates co-localization at the base of the photoreceptor connecting cilium. (**b’-d’**) are high magnification images of the boxed areas in (b-d). (**e-f”**) Centrosomal co-localization of NINL^isoB^, CC2D2A and MICAL3 in hTERT-RPE1 cells. mRFP-NINL^isoB^ (red signal, **e**) localizes to the basal body of the cilia marked with polyglutamylated tubulin (cyanid signal; **e**) and overlaps with GFP-tagged MICAL3 (green signal, **e’**) at the ciliary base when co-expressed (yellow signal, **e”**). Co-expression of eCFP-CC2D2A (green signal, **f**) and mRFP-MICAL3 (red signal, f’) resulted in partial overlap at the base of the cilia (yellow signal, **f”**). (**g**) Endogenous MICAL3 (green signal) detected by immunostaining clusters at the ciliary base (white arrows; cilium marked with anti-acetylated tubulin in red) of hTERT-RPE1 cells treated with non-targeting siRNA. (**h, i**) Knockdown of *NINL* (**h**) or *CC2D2A* (**i**) expression by siRNA results in dispersed distribution of MICAL3 throughout the cell body (brackets) with retention of some MICAL3 puncta at the ciliary base (arrows). qPCR analysis of *NINL* (**j**) and *CC2D2A* (**k**) siRNA treated hTERT-RPE1 cells. Cells were transfected with 10nM siRNA and all qPCR data were normalized against *GUSB* levels. Bar and error bars refer to mean and standard deviation, respectively (n = 3, on two biologicial replicates). *: P<0.05; **: P<0.01 versus non targeting siRNA (NT) (student’s *t-*test). Nuclei are counter stained with DAPI in all panels (blue signal). Scale bars: are 5 μm in d, 1 μm in d’ and 10 μm in e-i.

In order to unravel the underlying molecular cause of the observed vesicle accumulation and to identify proteins that interact with NINL, we next generated N-terminal Strep/FLAG-tagged fusion proteins of NINL isoA and isoB. A single-step affinity purification combined with quantification by stable isotope labeling of amino acids in cell culture (SILAC) and tandem affinity purification (TAP) [37] were applied to isolate the protein complexes in their native functional states from human embryonic kidney 293T (HEK293T) cells. The complexes were subsequently analyzed by liquid chromatography coupled with tandem mass spectrometry (LC-MS/MS). The identified interactome consisted of 174 unique proteins (Fig 6a, S1 table). An important association was found with multiple subunits of the cytoplasmic dynein 1-dynactin motor complex (DYNC1H1, DYNC1LI1, DYNC1LI2, DYNCI2, DYNLRB1, DCTN1-4, and DCTN6) which is involved in minus end–directed microtubule-associated transport. In addition, six actin-binding proteins (ARP1, ARP1B, ARP10, CAPZA1, CAPZA2 and CAPZB) and three subunits of Ca^2+^/calmodulin-dependent protein kinase II (CaMKII) (CAMK2A, CAMK2D, and CAMK2G), involved in non-canonical Wnt5a signaling, synaptic plasticity and kidney development [38], were found to associate with NINL. An additional relevant NINL interaction partner identified was MICAL3 (Microtubule-associated Monooxygenase, Calponin and LIM domain containing 3 protein), which is known to participate in a protein complex with RAB6 and RAB8 that is involved in the fusion of exocytotic vesicles [39], a process that appears to be deficient in the retina of *cc2d2a* mutants and *ninl* morphants. We validated the interaction between NINL^isoB^ and MICAL3 by reciprocal co-immunoprecipitations (Fig 6b) and confirmed that endogenously expressed MICAL3 is present at the photoreceptor connecting cilium in rat retina (P20), partially overlapping with the cilium and basal body marker polyglutamylated tubulin (Fig 7b–7d’). In hTERT-RPE1 cells, mRFP-tagged NINL^isoB^ (Fig 7e-e”) and eCFP-tagged CC2D2A (Fig 7f-f”) partially overlapped with tagged MICAL3.

To evaluate the role of NINL and CC2D2A in MICAL3 localization, we silenced the expression of *NINL* and *CC2D2A* in ciliated hTERT-RPE1 cells using siRNA, which was quantified by qPCR analysis (Fig 7j and 7k). Subsequent immunohistochemical stainings showed predominant MICAL3 localization at the ciliary base in non-targeting siRNA-treated cells (Fig 7g) whereas silencing of *NINL* expression resulted in a dispersed distribution of MICAL3 throughout the cell body (Fig 7h). Downregulation of *CC2D2A* expression in hTERT-RPE1 cells had a less pronounced effect on MICAL3 localization, resulting in partial mislocalization to the cell body (Fig 7i). These findings support a link between CC2D2A and MICAL3-RAB8-mediated vesicle trafficking/fusion through NINL.

## Discussion

**Fig 8:**
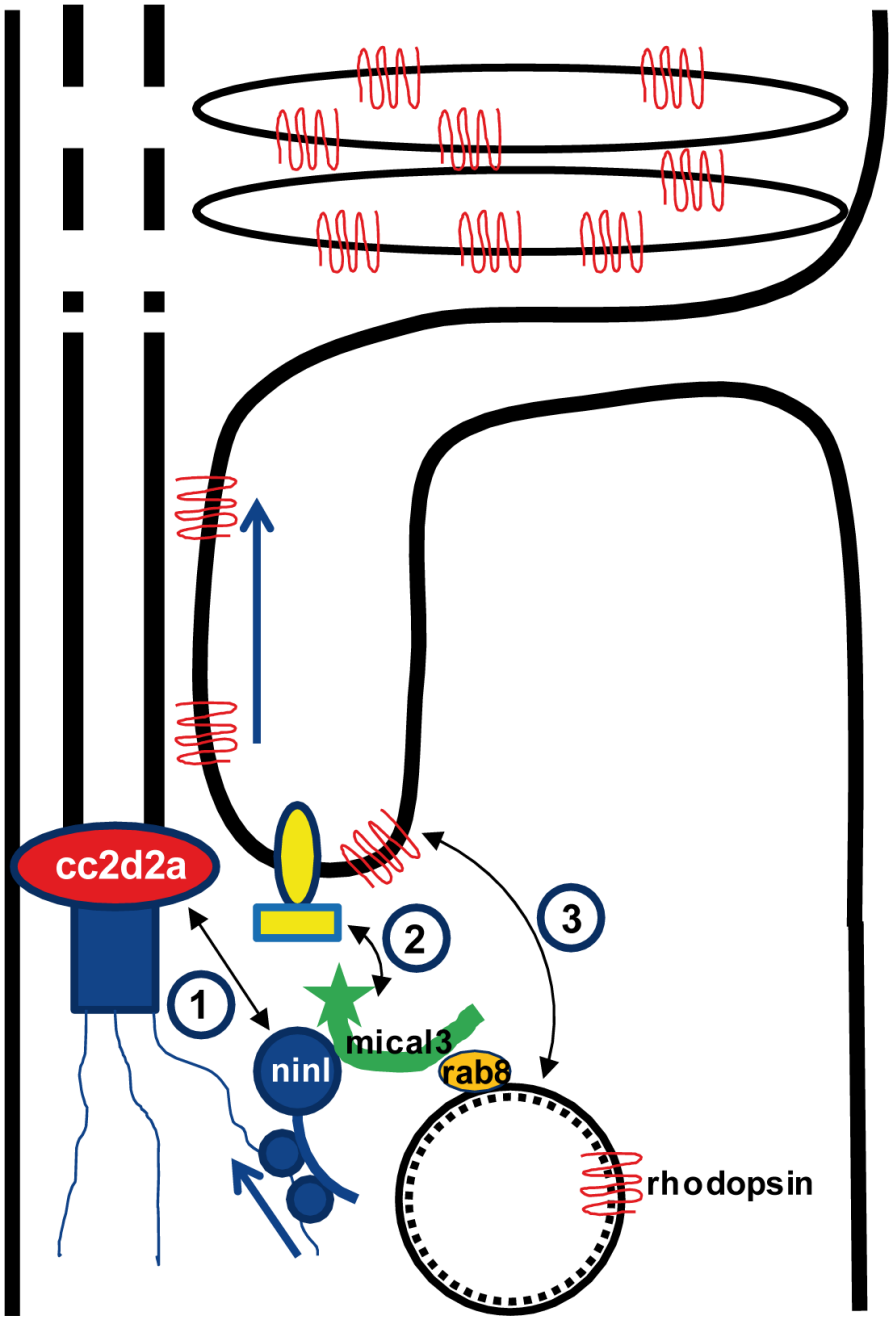
Proposed model for CC2D2A and NINL function in trafficking, docking and fusion of rhodopsin-carrier vesicles. 1) CC2D2A binds NINL and thus provides a docking point at the base of the connecting cilium for incoming vesicles. 2) NINL binds MICAL3 which in turn binds RAB8 that is coating the rhodopsin-carrier vesicles. Since NINL also associates with the cytoplasmic dynein1 motor complex, it provides a link between the carrier vesicles and the motor generating the movement along the microtubules. 3). MICAL3 subsequently interacts with ELKS and its redox activity promotes remodeling of the docking complex resulting in fusion of the vesicle at the periciliary region.

Dysfunction of transition zone proteins causes several ciliopathies such as Joubert syndrome, Meckel syndrome, nephronophthisis or Usher syndrome [16,21,40–43]. Previous work suggests that transition zone proteins in general, and CC2D2A in particular, are required for correct localization of transmembrane proteins to the ciliary membrane [7,25],. The mechanism by which transition zone proteins exert this function and the link to upstream ciliary-directed vesicular trafficking mechanisms remain however largely unknown. In this work, we identify NINL as a novel physical interaction partner for the transition zone protein CC2D2A and propose a model linking CC2D2A to RAB8A-controlled vesicle trafficking through a dual role for NINL in microtubule-based vesicle transport (Fig 8). The association of NINL with both the cytoplasmic dynein 1-dynactin motor complex (Dona et al, co-submitted manuscript) and MICAL3 supports a role for NINL in the initial transport of trans-Golgi network-derived RAB8A-MICAL3 coated vesicles towards the base of the photoreceptor cilium, while the association of NINL with CC2D2A provides a docking point for these incoming vesicles at the entrance of the ciliary compartment.

Ciliary transmembrane proteins are synthesized in the cell body and travel from the Golgi towards the cilium in vesicles which move along microtubules using a cytoplasmic dynein motor [44]. Once at the entrance of the ciliary compartment, these vesicles must dock and fuse with the periciliary membrane to deliver their cargo into the ciliary membrane [45]. This path has been particularly well studied in photoreceptors, where large quantities of opsins and membrane continuously have to replenish the disks which constitute this photo-sensitive structure [26,46,35]. Opsin trafficking is severely affected in both zebrafish *cc2d2a* mutants and *ninl* morphants, suggesting that both proteins play an important role in this transport which is crucial for the correct morphogenesis and homeostasis of the outer segments. Their co-localization at the base of the photoreceptor cilium could suggest that Cc2d2a and Ninl play a similar or combined role in opsin transport or that one protein is required to localize the other. However, since we found that each protein localizes independently of the other, and that *ninl* knockdown enhances the *cc2d2a* null mutant phenotype, the relationship between them is likely more complex than a simple linear pathway. At the ultrastructural level, the loss of function phenotypes of these two proteins also slightly diverge from each other: although vesicles accumulate in both cases in the affected photoreceptors, small vesiculo-tubular structures accumulate mostly apically around the connecting cilium in *cc2d2a* mutants [25], while this work shows that small vesicles and larger vacuoles are also present more basally and closer to an abnormal Golgi apparatus in *ninl* morphants. This suggests that both proteins are important for vesicular trafficking but play different roles in this process.

NINL has been previously shown to bind several other ciliopathy proteins present at the base of cilia, specifically LCA5 and USH2A [31], suggesting that it may play a more pivotal role in vesicular trafficking in photoreceptors than CC2D2A. Given that the zebrafish *ninl* morphant phenotype is more severe than the *cc2d2a* mutant phenotype, this further suggests a more central role for Ninl than for Cc2d2a in cilium-directed trafficking. This hypothesis is also supported by the lack of bi-allelic *NINL* mutations in a large human cohort of Joubert syndrome. Indeed, this may be interpreted as lack of tolerance to loss-of-function mutations in *NINL*, as these would lead to more severe phenotypes or early embryonic lethality. The direct interaction between NINL and the dynein 1-dynactin complex [47] which we confirmed and expanded in the associated study by Dona et al, suggests that NINL might be involved in minus end–directed microtubule-associated transport of organelles and cargo towards the base of the cilium. An appealing model would thus propose that NINL functions both more upstream in ciliary-directed vesicular trafficking than CC2D2A as well as at the base of the cilium where it interacts with several different proteins including CC2D2A.

While no bi-allelic rare deleterious *NINL* variants were identified in our JBTS cohort, we did find heterozygous *NINL* mutations in individuals with Joubert syndrome. Interestingly, only the individual with causal bi-allelic *CC2D2A* mutations and a heterozygous truncating *NINL* mutation had a severe phenotype with retinal and terminal renal disease. In comparison, the individuals with causal mutations in other JBTS genes and a heterozygous deleterious *NINL* mutation (or with the same causal *CC2D2A* mutation alone) had the classical “pure Joubert” phenotype without retinal or renal involvement. While bi-allelic *CC2D2A* mutations can result in a wide range of JBTS-associated phenotypes, the majority of individuals with causal *CC2D2A* mutations and JBTS display the “pure JBTS” phenotype [22]. The more severe phenotype only of the individual carrying causal *CC2D2A* mutations and an additional *NINL* truncating variant suggests that deleterious variants in *NINL* may act as genetic modifiers specifically of *CC2D2A*-caused ciliopathies such as Joubert syndrome. Unfortunately, the rarity of this disorder and its prominent genetic heterogeneity with over 27 associated genes prevent identification of multiple individuals sharing the same combination of causal and additional genetic variants, precluding identification of a statistically significant effect of rare variants as genetic modifiers using human genetics alone. Our findings from a large Joubert cohort therefore remain of anecdotal nature. However, the physical and genetic interaction in zebrafish identified in this work substantially strengthen the significance of this finding and suggest that deleterious variants in *NINL* may indeed enhance the retinal and renal phenotype in individuals with *CC2D2A*-associated Joubert syndrome. The effect on the retinal phenotype may be explained by the importance of NINL function in photoreceptors as highlighted in the present study. Enhancement of the renal phenotype by the additional *NINL* mutation may be explained by the association identified in this study between NINL and the PKD2-target CaMKII, which is important for renal development [38].

The identification of MICAL3 as an interaction partner for NINL is of particular relevance in the context of vesicular trafficking given that MICAL3 binds RAB8A and plays a role in exocytotic vesicle fusion [39]. MICAL3 is part of the MICAL family of flavoprotein monooxygenases which regulate the actin cytoskeleton by disassembling the actin filaments. The redox function of MICAL3 is required to promote vesicle fusion, possibly by destabilizing protein complexes and remodeling the docking-fusion complexes in which it is engaged [39]. The role of RAB8A in vesicle fusion at the ciliary base has been abundantly documented in various cell types including photoreceptors [27,28]. While RAB8A was found to bind several ciliopathy proteins directly including CEP290 and RGPR [48,49], no direct interaction has been demonstrated between CC2D2A and RAB8A, despite a functional interaction in zebrafish photoreceptors and a requirement for CC2D2A in RAB8A localization in mouse embryonic fibroblasts [24,25]. Our findings now provide a model explaining the link between CC2D2A and RAB8A (Fig 8): RAB8A-coated vesicles destined to the ciliary compartment are bound by MICAL3 which in turn binds NINL that is associated to the cytoplasmic dynein 1 motor complex (Dona et al, companion manuscript), allowing the movement along the microtubules. Once at the base of the cilium, NINL interacts with CC2D2A, providing the specificity of the docking point at the entrance to the ciliary compartment. Finally, the redox activity of MICAL3 promotes remodeling of the complex allowing fusion of the vesicle and release of cargo into the peri-ciliary membrane.

A role for CC2D2A in promoting the assembly of ciliary subdistal appendages was recently suggested whereby CC2D2A would be required for docking of transport vesicles [24]. This is compatible with our model which also provides a possible mechanism to explain how transition zone proteins may regulate ciliary protein composition by providing specific docking points at the entry to the ciliary compartment. Dysfunction of transition zone proteins can lead to a variety of ciliopathies and it is likely that abnormal ciliary protein composition is at least in part responsible for the observed disease phenotypes even in the absence of ciliogenesis defects. This provides an opportunity for the development of pathway-specific therapies aiming at modulating trafficking routes and restoring normal ciliary protein content. In this perspective, unraveling the cell biological function of disease genes such as *CC2D2A* as presented in the current study is a prerequisite for the future development of pharmacological treatments for patients with ciliopathies.

## Materials and Methods

### Ethics statement

All animal protocols were in compliance with Swiss legal ethical guidelines and the European Union Regulatory Agency guidelines for the use of fish in biomedical research and experiments and were approved by the local authorities (Veterinäramt Zürich TV4206). Human Subject Research Procedures were approved by the Institutional Review Boards at the University of Washington and Seattle Children’s Hospital (IRB-UW # 28853), and all participants or their legal representatives provided written informed consent.

### Zebrafish

Zebrafish (*Danio Rerio*) were maintained as described [50]. The *cc2d2a* ^*w38*^ */sentinel* mutant (referred to as *cc2d2a* mutant or *cc2d2a*-/-) was previously described [25,51,52]. The transgenic Tg(wt1b:EGFP) line was previously described [53]. Embryos were raised at 28°C in embryo medium and pigment development was inhibited by phenylthiourea as described in Westerfield [50]. *ninl* translation-blocking (5’-CATCCTCGTCCATCCCACCACATAC-3’) morpholino (MO) and splice blocking (5’-CCCAACACTAAAGAGATACACCAAT-3’) morpholinos were designed by Gene Tools Inc. (USA) and 1nl was injected into zebrafish embryos at the one-cell stage. After a titration curve, we established that 2ng/nl was the optimal phenotypic dose consistently causing the major phenotypes without significant cell death, while at the low dose of 0.75ng/nl, no phenotypes were observed (therefore called the “sub-phenotypic dose”). For the splice morpholino, the optimal phenotypic dose was 4ng/nl. For rescue experiments, cDNA encoding full length human *NINL* isoform B was cloned into a pCS2+ vector made compatible with the Gateway system (Invitrogen, USA), pCS2+/DEST, and subsequently transcribed with the SP6 Message Machine kit (Ambion, USA) according to manufacturer’s instructions. The cherry-Rab8a construct was previously described [25]. All quantifications were performed blinded as to injection status. All animal protocols were in compliance with internationally recognized guidelines for the use of fish in biomedical research and experiments and were approved by the local authorities (Veterinäramt Zürich TV4206).

### Plasmids

pDONR201 vectors containing cDNA encoding human NINL isoform A and B as well as aa 1–998, aa 433–637, aa 992–1177 of human *CC2D2A* were previously described [31,52]. Using Gateway cloning technology, cDNA fragments encoding aa 992–1620 and aa 1171–1620 of human *CC2D2A* (NM_001080522) were cloned in pDONR201 according to manufacturer’s instructions. pEGFP-C1-MICAL3 was kindly provided by Dr. A. Akhmanova (Utrecht University, The Netherlands).

### Yeast two-hybrid interaction assay

The direct interaction between CC2D2A and other ciliary proteins was tested using a GAL4-based yeast two-hybrid system (Hybrizap, Stratagene, USA) as previously described [30]. The DNA binding domain (GAL4-BD) fused to full length CC2D2A was used as a bait to test the interaction with previously described ciliopathy and cilium-associated proteins fused to an activation domain (GAL4-AD). Constructs encoding GAL4-BD and GAL4-AD fusion proteins were co-transformed in yeast strain PJ69-4A. The direct interaction between baits and preys induced the activation of the reporter genes, resulting in the growth of yeast colonies on selective media (deficient of histidine and adenine) and induction of α-galactosidase and β-galactosidase colorimetric reactions [54].

### Affinity purification of protein complexes

HEK293T cells transiently expressing the SF-TAP tagged NINL^isoB^ were grown in SILAC DMEM (PAA) supplemented with 3 mM l-glutamine (PAA), 10% dialyzed fetal bovine serum (PAA), 0.55 mM lysine, and 0.4 mM arginine. Light SILAC medium was supplemented with ^12^C_6_,^14^N_2_ lysine and ^12^C_6_,^14^N_4_ arginine. Heavy SILAC medium was supplemented with either ^13^C_6_ lysine and ^13^C_6_,^15^N_4_ arginine or ^13^C_6_,^15^N_2_ lysine and ^13^C_6_,^15^N_4_ arginine. 0.5 mM proline was added to all SILAC media to prevent arginine-to-proline conversion [55]. All amino acids were purchased from Silantes. For one-step Strep purifications, SF-TAP–tagged proteins and associated protein complexes were purified essentially as described previously [37,56]. HEK293T cells transiently expressing the SF-TAP tagged constructs were lysed in lysis buffer containing 0.5% Nonidet-P40, protease inhibitor cocktail (Roche), and phosphatase inhibitor cocktails I and II (Sigma-Aldrich) in TBS (30 mM Tris-HCl, pH 7.4, and 150 mM NaCl) for 20 minutes at 4°C. After sedimentation of nuclei at 10,000 *g* for 10 minutes, the cleared lysates were transferred to Strep-Tactin-Superflow beads (IBA) and incubated for 1 hour before the resin was washed 3 times with wash buffer (TBS containing 0.1% NP-40 and phosphatase inhibitor cocktails I and II). The protein complexes were eluted by incubation for 10 minutes in Strep-elution buffer (IBA). After purification, the samples were precipitated with chloroform and methanol and subjected to in-solution tryptic cleavage as described previously [57]. LC-MS/MS analysis was performed on an Ultimate3000 nano HPLC system (Dionex) coupled to a LTQ OrbitrapXL mass spectrometer (Thermo Fisher Scientific) by a nanospray ion source. The raw data were analyzed using Sequest (Thermo Fisher Scientific) or Mascot and Scaffold (Proteome Software) as described previously [57]. Proteins were considered to be specific protein complex components if they were not detected in the control and were detected at least twice with two or more peptides (peptide probability >80%) in three experiments. The protein probability threshold was set to 99%.

### Knockdown of *NINL* and *CC2D2A* in cultured hTERT-RPE1 cells by RNAi

Three Silencer Select siRNAs targeting *NINL* and *CC2D2A* were purchased from Life Technologies (targeting sequences are listed in S2 Table). For transfection, a pool of three siRNAs per gene (45 nM final concentration) were plated in MW12 plates with or without glass slides. Lipofectamine RNAiMax (LifeTechnologies) and Opti-MEM (LifeTechnologies) were added to the duplexes and incubated for 10–20 minutes according to manufacturer’s protocol to allow the formation of transfection complexes. Human telomerase reverse transcriptase-transformed retinal pigment epithelium (hTERT-RPE1) cells from American Type Culture Collection (ATCC) were then plated in MW12 plates. Per plate, non-targeting Silencer Select duplexes (LifeTechnologies) were included as negative controls. After 24 hours of transfection, cells were serum-starved to induce ciliogenesis. After 72 hours of transfection, knockdown-efficiency was determined by isolating total RNA from one 12-well with Trizol (Invitrogen, USA), followed by first-strand cDNA synthesis (iScript; Bio-Rad, USA). Quantitative PCRs using GoTaq (Promega), with validated *NINL-*, *CC2D2A-* and *GUSB*-specific primers (sequences are listed in S3 Table), were performed as previously described [31]. The second 12-well of cells were fixed with 2% paraformaldehyde, permeabilized with 1% Triton-X-100/PBS and stained with anti-MICAL3 antibodies (kindly provided by Dr. A. Akhmanova). Images were taken with an Axioplan2 Imaging fluorescence microscope (Zeiss, Germany) equipped with a DC350FX camera (Zeiss, Germany).

### Co-immunoprecipitation in HEK293T cells

HA-tagged NINL isoform B was expressed by using the mammalian expression vector pcDNA3-HA/DEST, FLAG-tagged CC2D2A, LRRK2 and STRAD by using p3xFLAG-CMV/DEST and strep-FLAG-tagged NINL isoform B by using pNTAPe5/DEST from the Gateway cloning system (Invitrogen, USA). eGFP and eGFP-tagged MICAL3 were expressed from pEGFP-C1 (Clontech, USA). All plasmids contain a CMV promoter. HEK293T cells were co-transfected using Effectene (Qiagen, USA) according to manufacturer’s instructions. Twenty-four hours after transfection cells were washed with PBS and subsequently lysed on ice in lysis buffer (50 mM Tris-HCl pH 7.5, 150 mM NaCl, 1% Triton-X-100 supplemented with complete protease inhibitor cocktail (Roche, Germany)). HA-tagged NINL isoform B was immunoprecipitated from cleared lysates overnight at 4°C by using rat monoclonal anti-HA-beads (Roche, Germany), while FLAG-tagged CC2D2A, LRRK2, STRAD and NINL isoform B were immunoprecipitated by using monoclonal anti-FLAG M2 Agarose beads (Sigma, Germany) and eGFP-tagged MICAL3 was immunoprecipitated using anti-GFP polyclonal antibodies (Abcam) coupled to ProtA/G beads (Santa Cruz, USA). After 4 washes in lysis buffer, the protein complexes were analyzed on immunoblots using the Odyssey Infrared Imaging System (LI-COR, USA). Tagged molecules were detected by anti-HA, anti-FLAG or anti-GFP mono- or polyclonal antibodies. As secondary antibody IRDye800 goat-anti-mouse IgG (Rockland Antibodies and Assays) and Alexa Fluor 680 goat-anti-rabbit IgG (Life Technologies) were used.

### Immunohisto- and immunocytochemistry

Zebrafish larvae were fixed in 4% paraformaldehyde (PFA) overnight at 4°C, embedded in OCT and cryosectioned following standard protocols. Sections were blocked using PBDT (PBS, 1% DMSO, 0.1% Triton X, 2mg/ml BSA) with 10% goat serum for 30 minutes at RT before incubation with primary antibodies overnight. Primary antibodies were mouse monoclonal anti-acetylated alpha tubulin (1:500, clone 6-11B-1, Sigma), mouse monoclonal anti-polyglutamylated tubulin GT335 (1:500, gift from C. Janke, Institut Curie, France), mouse anti-zebrafish Cc2d2a (1:20, [25]), rabbit anti-NINL (1:100; LSBio Cat# LS-C201509), mouse anti-*pan* centrin 20H5 (1:200, clone 20H5 Millipore), mouse anti-Rab8a (1:100, clone 3G1 Novus Biologicals), mouse anti-opsin 4D2 (1:100, gift from R. Molday, University of British Columbia) and rabbit anti-Ift88 (gift from B. Perkins [58], Cleveland Clinic), mouse monoclonal anti-FLAG (1:1000, Sigma), rabbit polyclonal anti-human MICAL3 [39]. Secondary antibodies were Alexa Fluor goat anti-rabbit or goat anti-mouse IgG (Life Technologies) used at 1:300. Bodipy (1:300, Invitrogen) was applied for 20 minutes after the secondary antibodies and nuclei were counterstained with DAPI. Rab8 puncta detected by immuno-staining using the mouse anti-Rab8 antibody were analyzed blinded as to injection status in ImageJ. A region of interest was manually determined on single confocal sections and was thresholded (allways with the same parameters); the “analyze particles” function of ImageJ was then used to determine the number of puncta per μm^2^. For quantification of intracellular fluorescence after 4D2 (opsin) immuno-staining, a region of interest including 10–15 photoreceptor cell bodies was determined on single confocal sections using ImageJ and the mean grey value was measured. For quantification of the proximal pronephric area, the fluorescent region corresponding to the glomerulus and the proximal tubules up to the curved part of the tubule was outlined manually in ImageJ and the “measure” function was used to determine the area of the outlined region. All quantifications were performed blinded as to injection status. Confocal imaging was performed on a Leica HCS LSI.

### Paraffin sections and Transmission Electron Microscopy

For paraffin sections, 4 dpf old *ninl* morphant larvae were fixed in 4% PFA overnight at 4°C, embedded in paraffin and sectioned following standard protocols. For Transmission Electron Microsopy, *ninl* morphant and control larvae were fixed overnight at 4°C in a freshly prepared mixture of 2,5% glutaraldehyde and 2% paraformaldehyde in 0.1 M sodiumcacodylate buffer (pH 7.4). After rinsing in buffer, specimens were post-fixed in a freshly prepared mixture, containing 1% osmiumtetroxide and 1% potassiumferrocyanide in 0.1 M sodiumcacodylate buffer (pH 7.4), during 2 h at room temperature. After rinsing, tissues were dehydrated through a graded series of ethanol and embedded in epon. Ultrathin (rostrocaudally) sections (70nm), comprising zebrafish eyes at the optic nerve level, were collected on formvar coated grids, subsequently stained with 2% uranyl acetate and Reynold’s lead citrate, and examined with a Jeol1010 electron microscope.

### Sequencing of *NINL* in a cohort of Joubert syndrome patients

346 individuals (from 291 families) with Joubert syndrome (JBTS) from the University of Washington Joubert Research Center were examined for mutations in *NINL*. Minimal enrollment criteria included clinical findings of JBTS (intellectual impairment, hypotonia, ataxia) and diagnostic or supportive brain imaging findings, or presence of a sibling with JBTS along with supportive clinical or imaging features. Procedures were approved by the Institutional Review Boards at the UW and Seattle Children’s Hospital, and all participants or their legal representatives provided written informed consent. Genomic DNA from peripheral blood or saliva was extracted and all *NINL* exons were captured by Molecular Inversion Probes (MIPS) [33]. Captured DNA was PCR amplified and sequenced on either the Illumina HiSeq or MiSeq platform. Sequence reads were mapped using the Burrows-Wheeler Aligner (BWA v.0.5.9). Variants were called using the Genome Analysis Tookit (GATK v2.5–2) and annotated with SeattleSeq (http://snp.gs.washington.edu/SeattleSeqAnnotation138/). Minimal quality criteria for analyzed variants were DP (Depth) ≥ 8, QD (Quality by Depth) > 5, and ABHet (Heterozygous Allele Balance) <0.8. The variant list was then filtered for rare and deleterious variants. Only variants with minor allele frequency of <1% were considered given the rarity of JBTS (estimated prevalence 1/80’000 [11]). All non-sense, frameshift and canonical splice-site variants, as well as missense variants with Polyphen2 scores >0.8 were considered deleterious. Selected variants were Sanger confirmed.

### Statistical analyses

For all quantifications of zebrafish experiments, the Graphpad Prism6 software (http://www.graphpad.com/scientific-software/prism/) was employed to generate scatter plots, calculate mean values and SEM values, and perform statistical tests. Continuous data was analyzed using two-tailed, unpaired Student’s t-test and categorical data was analyzed using Fisher’s exact test.

## Supporting Information

S1 Fig Cloning and characterization of zebrafish *ninl*. (**A**) Schematic representation of the protein structure of human NINL^isoA^ and NINL^isoB^ (*H*.*s*. NINL^isoA^ and *H*.*s*. NINL^isoB^) and zebrafish ninl (*D*.*r*. ninl) as predicted by using the Pfam homepage (http://pfam.xfam.org/). (**B**) *Ninl* expression during zebrafish development by whole mount RNA *in situ* hybridization. Specific expression was found in the following structures as indicated by numbers and arrows: (**a**) 14 somite stage: otic placode (1); developing eye (2); neural tube (spinal cord) (3); (**b**) 18 somite stage: neural tube (3); pronephros (4); (**c**) 18 somite stage: inner ear (1); optic nerve (5). (**d**) At 6 dpf, expression was observed in the tectum (6), the heart (7) and in the eye (2), predominantly in the photoreceptor cell layer. *H*.*s*.: *homo sapiens*; *D*.*r*.: *danio rerio*; CC: coiled-coil; IF: intermediate filament domain; som: somites; dpf: days post-fertilization.
(TIF)

S2 Fig Specificity of the anti-Ninl antibody. (**a**) Indirect immunohistochemical staining using anti-Ninl antibody on 4 dpf retinal cryosections of control MO-injected larvae (green signal) shows punctate staining, partially overlapping with the ciliary marker anti-polyglutamylated tubulin (**a’, a”**, red signal). (**b**) Indirect immunohistochemical staining using anti-Ninl antibody on 4 dpf retinal cryosections of *ninl* atgMO-injected larvae (**b**, green signal) along with the ciliary marker anti-polyglutamylated tubulin (**b’, b”**, red signal). Specific Ninl-immunofluorescence is largely abolished in *ninl* morphants, whereas the polyglutamylated tubulin signal is still detected. (**c**) Indirect immunohistochemical staining of anti-Ninl on 4 dpf retinal cryosections of *ninl* ex15 spMO-injected larvae (green signal) along with the ciliary marker anti-polyglutamylated tubulin (**c’, c”**, red signal). Specific Ninl-immunofluorescence was still detected but at a diminished level in *ninl* morphants whereas the polyglutamylated tubulin signal was unaltered. Nuclei are stained with DAPI (blue signal). Scale bars: 4 μm. (**d**) Western blot analysis using protein extracts obtained from 100 zebrafish larvae injected with either control MO (6ng), *ninl* atgMO (2ng) or *ninl* ex15 spMO (4ng). A specific product was detected with a molecular weight of ~80kDa in control MO-injected larvae (left panel). This band was almost completely abolished in the *ninl* atgMO-treated larvae, but was still detected in *ninl* spMO-injected larvae although with a slightly diminished intensity. Anti-actin antibodies were used as a loading control (right panel). (**e**) Immunoprecipitation from bovine retinal extracts with anti-human NINL antibody detects 3 bands, the strongest being of the same size as the band found on Western blot of zebrafish lysates (~80 kDa).
(TIF)

S3 Fig Phenotypes of the *ninl* atgMO. (**a**) Representative clutch of zebrafish larvae at 2dpf injected with the phenotypic dose of *ninl* atgMO (2ng/nl). (b) Titration curve for the *ninl* atgMO illustrating the distribution of phenotypes in 2dpf larvae at two different concentrations: at 1ng/nl, a minority of injected larvae present a curved body shape (10%) and/or ventriculomegaly (20%) (n = 19). At ~2ng/nl, on average 66% of injected larvae present a curved body shape and ~40% present ventriculomegaly and/or pronephric cysts (n = 84). 95% Confidence Interval bars are shown. (c) Representative 2dpf-old *ninl* atg-morphant displaying curved body shape, ventriculomegaly and pronephric cyst (arrow). (**d-e**) Dorsal view of 2dpf larvae showing the normal morphology of the brain folds in wild-type (**d**) and the enlarged ventricle in morphants (**e**). (**f-g**) Transgenic Tg(wt1b:EGFP) zebrafish line used to highlight the larval pronephros, shows the morphology of the fused glomerulus and proximal tubules in wild-type (**f**) and the dilatation of the region in *ninl* morphants (“kidney cysts”, white arrow in **g**). (**h**) A clutch of *ninl* morphants at 4dpf. (**i-j**) TUNEL assay on 4dpf cryosections through retinas from *ninl* morphants shows the range of cell death detected (curved larvae in (h) were sectioned for the TUNEL assay). Note the absence of TUNEL-positive cells in the photoreceptor (PR) cell layer in the morphants. (**k**) The *ift88*-/- retina is used as a positive control, given the known death of photoreceptors in this mutant at 4dpf. (**l**) Cryosection through a 4dpf brain in a morphant larva displaying a dilated brain ventricle (v) shows no significant neuronal cell death. Scale bars represent 500 μm in (a) and (h), 100 μm in (c-g), 10 μm in (i-k) and 30 μm in (l). PR PhotoReceptors, INL Inner Nuclear Layer.
(TIF)

S4 Fig Rescue of the morphant phenotype supports its specificity. (**a-d**) Co-injection of 2 ng *ninl* atgMO with 150 pg capped MO-resistant mRNA encoding human *NINL* isoform B reduced the incidence of body curvature defects from 71% in *ninl* atgMO injected larvae (n = 207) to 36% in *ninl* atgMO + *NINL* mRNA injected larvae (n = 203) (*P*<0.0001, two-tailed Fisher’s exact). A subset of these larvae were sectioned and a perfect correlation was observed in rescue between body curvature defects and defects in photoreceptor outer segment formation (**a’-c’**). (**e**) Quantification of the rescue of retinal outer segment length showing that mean OS length was rescued from 1.6 +/- 0.26 μm in *ninl* morphants to 3.8 +/- 0.25 μm with co-injection of *NINL* mRNA (P<0.0001, unpaired Student’s *t*-test).
(TIF)

S5 Fig Recapitulation of phenotype by a *ninl* ex15 spMO (4 dpf). (**a-b**) Injection of 4ng *ninl* ex15 spMO (n>100) results in heart edema and small eyes. No defects in body curvature were observed in comparison to control MO-injected larvae. (**a’, b’**) Analyses of bodipy-stained retinas of *ninl* ex15 spMO-injected larvae (n = 10) revealed defects in photoreceptor outer segment formation (10 of 10) similar to those observed in *ninl* atgMO-treated larvae, whereas stained retinas of control MO-injected larvae (n = 10) appeared normal (10 of 10). Scale bars represent 500μm (a-b) and 5μm (a’-b’). (**c**) RT-PCR analysis on RNA isolated from 25 larvae that were either uninjected, injected with control MO (6ng) or injected with various amounts of *ninl* ex15 spMO (2, 4, 6ng), collected at two different time points after injection (2 dpf and 4 dpf). One PCR product of the expected length (~500bp) was obtained from RNA from uninjected and control MO-injected larvae. Sequence analysis revealed that this was the predicted transcript including exons 13–16. RT-PCR analysis on RNA obtained from the morphant larvae resulted in two products: Sequence analysis of both fragments revealed that the shorter product is the predicted wild-type transcript (ex13-16) and that the longer transcript in addition includes the entire intron 14 (85 bp), resulting in premature termination of translation already after two codons in intron 14. This aberrant splicing persists at 4dpf.
(TIF)

S1 Table TAP-data and SILAC data. **SF-TAP analysis with over-expressed N-terminally SF-TAP-tagged NINL in HEK293T cells.** Shown are the number of unique identified peptides as well as the sequence coverage for each protein detected by mass spectrometry. Proteins identified in the SF-TAP analysis of empty vector control experiments were removed. **SILAC analysis with over-expressed N-terminally SF-TAP-tagged NINL in HEK293T cells**. Shown are the ratios and significance value for WT/SF-control experiments.
(XLSX)

S2 Table siRNA sequences. (DOCX)

S3 Table Primer sequences. (DOCX)
