# Supplementary figures and images for: The Ciliopathy Protein CC2D2A Associates with NINL and Functions in RAB8-MICAL3-Regulated Vesicle Trafficking
Source: PLoS Genet. 2015 Oct 20;11(10):e1005575. doi: 10.1371/journal.pgen.1005575 (PMC4617701; doi:10.1371/journal.pgen.1005575)

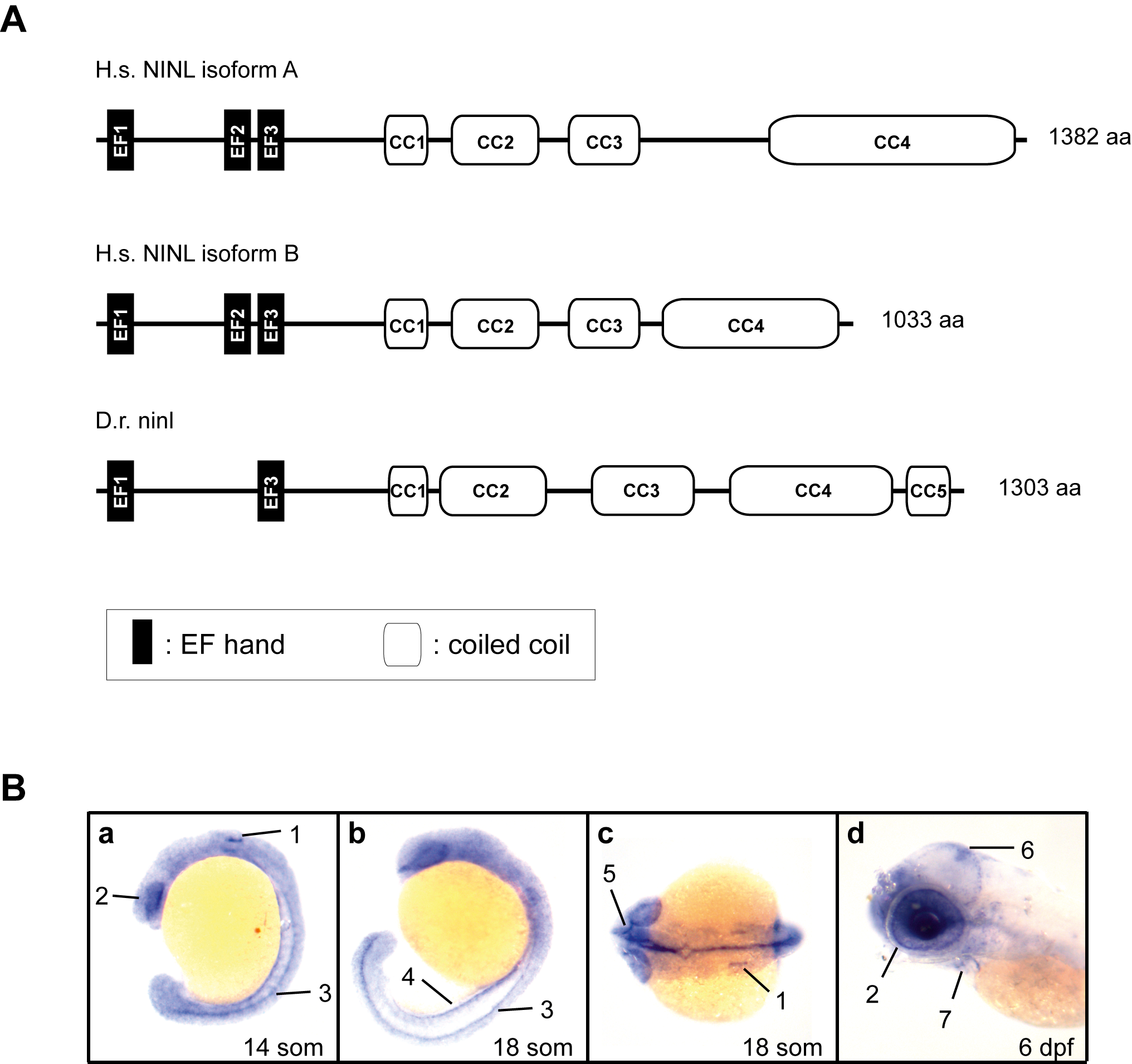

Supplement: S1 Fig — (A) Schematic representation of the protein structure of human NINLisoA and NINLisoB (H.s. NINLisoA and H.s. NINLisoB) and zebrafish ninl (D.r. ninl) as predicted by using the Pfam homepage (http://pfam.xfam.org/). (B) Ninl expression during zebrafish development by whole mount RNA in situ hybridization. Specific expression was found in the following structures as indicated by numbers and arrows: (a) 14 somite stage: otic placode (1); developing eye (2); neural tube (spinal cord) (3); (b) 18 somite stage: neural tube (3); pronephros (4); (c) 18 somite stage: inner ear (1); optic nerve (5). (d) At 6 dpf, expression was observed in the tectum (6), the heart (7) and in the eye (2), predominantly in the photoreceptor cell layer. H.s.: homo sapiens; D.r.: danio rerio; CC: coiled-coil; IF: intermediate filament domain; som: somites; dpf: days post-fertilization. (TIF) [file pgen.1005575.s001.tif]

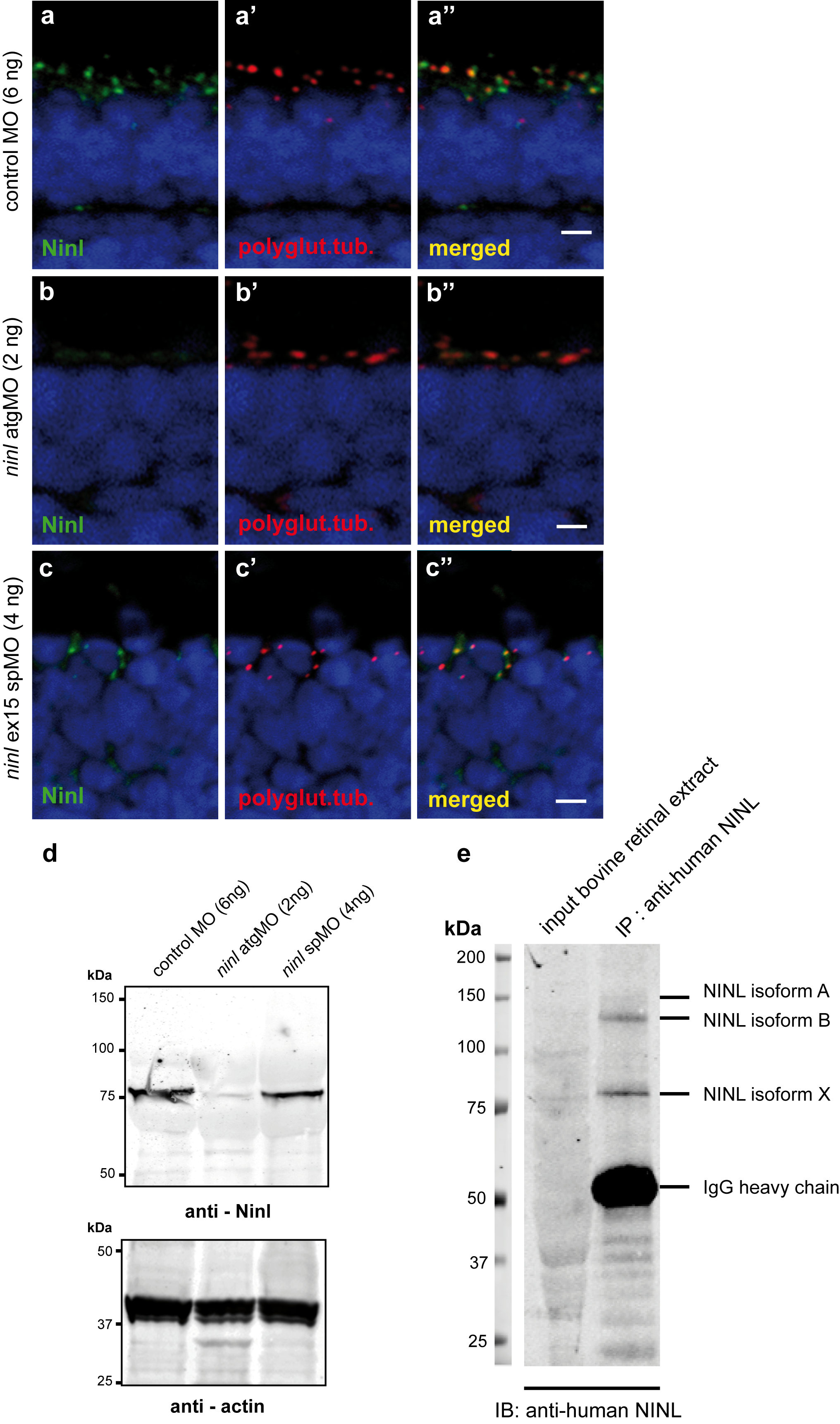

Supplement: S2 Fig — (a) Indirect immunohistochemical staining using anti-Ninl antibody on 4 dpf retinal cryosections of control MO-injected larvae (green signal) shows punctate staining, partially overlapping with the ciliary marker anti-polyglutamylated tubulin (a’, a”, red signal). (b) Indirect immunohistochemical staining using anti-Ninl antibody on 4 dpf retinal cryosections of ninl atgMO-injected larvae (b, green signal) along with the ciliary marker anti-polyglutamylated tubulin (b’, b”, red signal). Specific Ninl-immunofluorescence is largely abolished in ninl morphants, whereas the polyglutamylated tubulin signal is still detected. (c) Indirect immunohistochemical staining of anti-Ninl on 4 dpf retinal cryosections of ninl ex15 spMO-injected larvae (green signal) along with the ciliary marker anti-polyglutamylated tubulin (c’, c”, red signal). Specific Ninl-immunofluorescence was still detected but at a diminished level in ninl morphants whereas the polyglutamylated tubulin signal was unaltered. Nuclei are stained with DAPI (blue signal). Scale bars: 4 μm. (d) Western blot analysis using protein extracts obtained from 100 zebrafish larvae injected with either control MO (6ng), ninl atgMO (2ng) or ninl ex15 spMO (4ng). A specific product was detected with a molecular weight of ~80kDa in control MO-injected larvae (left panel). This band was almost completely abolished in the ninl atgMO-treated larvae, but was still detected in ninl spMO-injected larvae although with a slightly diminished intensity. Anti-actin antibodies were used as a loading control (right panel). (e) Immunoprecipitation from bovine retinal extracts with anti-human NINL antibody detects 3 bands, the strongest being of the same size as the band found on Western blot of zebrafish lysates (~80 kDa). (TIF) [file pgen.1005575.s002.tif]

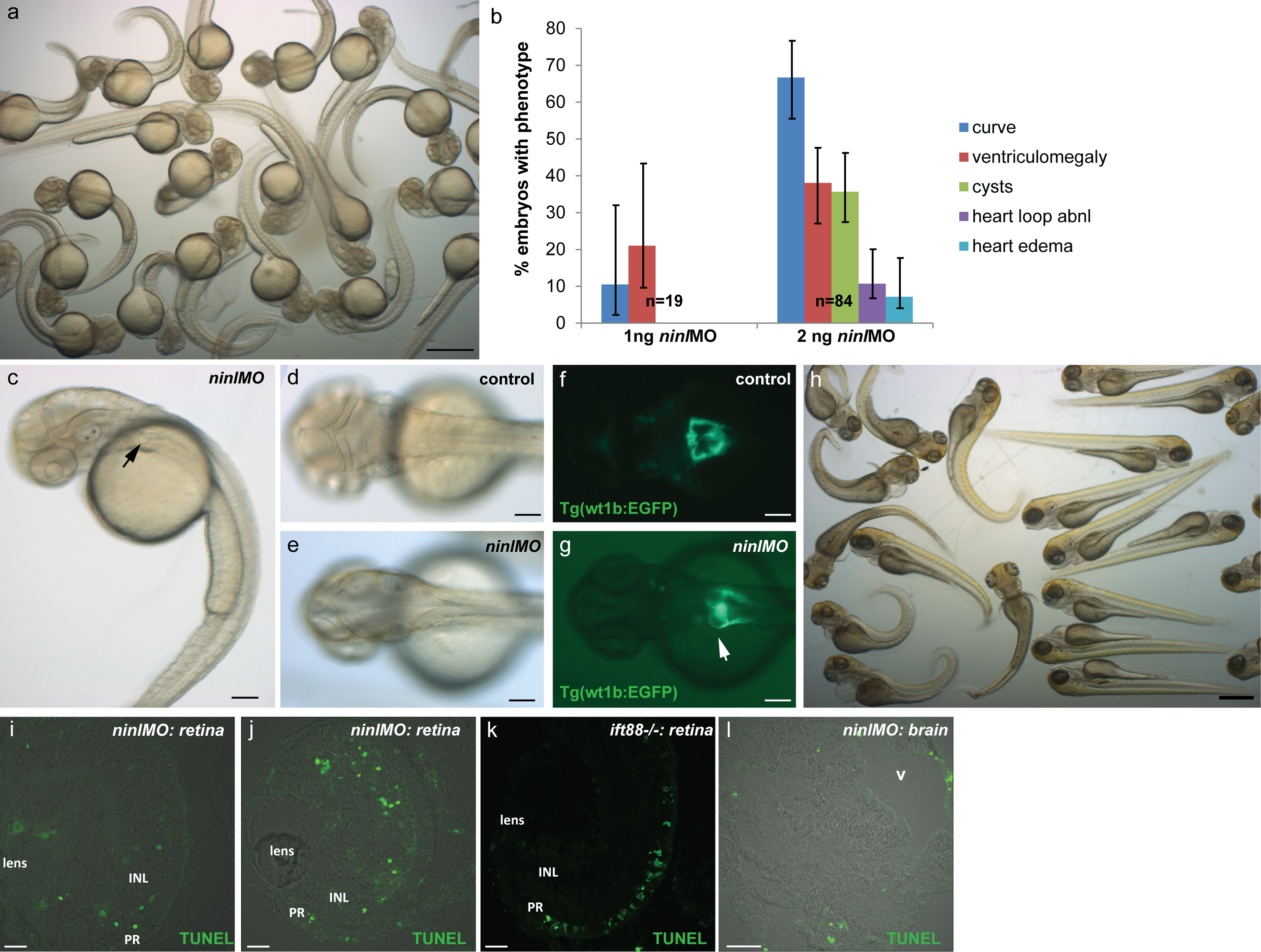

Supplement: S3 Fig — (a) Representative clutch of zebrafish larvae at 2dpf injected with the phenotypic dose of ninl atgMO (2ng/nl). (b) Titration curve for the ninl atgMO illustrating the distribution of phenotypes in 2dpf larvae at two different concentrations: at 1ng/nl, a minority of injected larvae present a curved body shape (10%) and/or ventriculomegaly (20%) (n = 19). At ~2ng/nl, on average 66% of injected larvae present a curved body shape and ~40% present ventriculomegaly and/or pronephric cysts (n = 84). 95% Confidence Interval bars are shown. (c) Representative 2dpf-old ninl atg-morphant displaying curved body shape, ventriculomegaly and pronephric cyst (arrow). (d-e) Dorsal view of 2dpf larvae showing the normal morphology of the brain folds in wild-type (d) and the enlarged ventricle in morphants (e). (f-g) Transgenic Tg(wt1b:EGFP) zebrafish line used to highlight the larval pronephros, shows the morphology of the fused glomerulus and proximal tubules in wild-type (f) and the dilatation of the region in ninl morphants (“kidney cysts”, white arrow in g). (h) A clutch of ninl morphants at 4dpf. (i-j) TUNEL assay on 4dpf cryosections through retinas from ninl morphants shows the range of cell death detected (curved larvae in (h) were sectioned for the TUNEL assay). Note the absence of TUNEL-positive cells in the photoreceptor (PR) cell layer in the morphants. (k) The ift88-/- retina is used as a positive control, given the known death of photoreceptors in this mutant at 4dpf. (l) Cryosection through a 4dpf brain in a morphant larva displaying a dilated brain ventricle (v) shows no significant neuronal cell death. Scale bars represent 500 μm in (a) and (h), 100 μm in (c-g), 10 μm in (i-k) and 30 μm in (l). PR PhotoReceptors, INL Inner Nuclear Layer. (TIF) [file pgen.1005575.s003.tif]

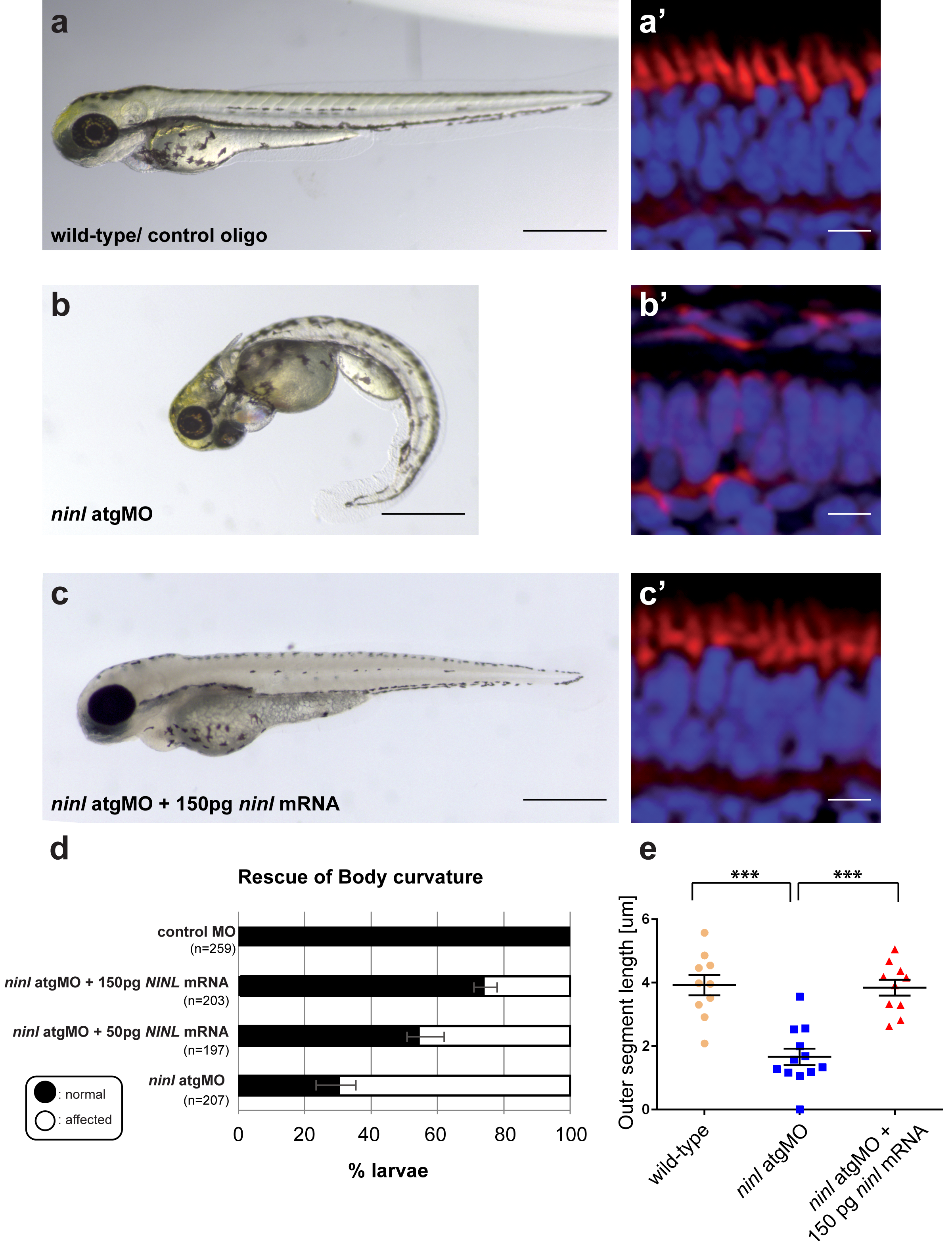

Supplement: S4 Fig — (a-d) Co-injection of 2 ng ninl atgMO with 150 pg capped MO-resistant mRNA encoding human NINL isoform B reduced the incidence of body curvature defects from 71% in ninl atgMO injected larvae (n = 207) to 36% in ninl atgMO + NINL mRNA injected larvae (n = 203) (P<0.0001, two-tailed Fisher’s exact). A subset of these larvae were sectioned and a perfect correlation was observed in rescue between body curvature defects and defects in photoreceptor outer segment formation (a’-c’). (e) Quantification of the rescue of retinal outer segment length showing that mean OS length was rescued from 1.6 +/- 0.26 μm in ninl morphants to 3.8 +/- 0.25 μm with co-injection of NINL mRNA (P<0.0001, unpaired Student’s t-test). (TIF) [file pgen.1005575.s004.tif]

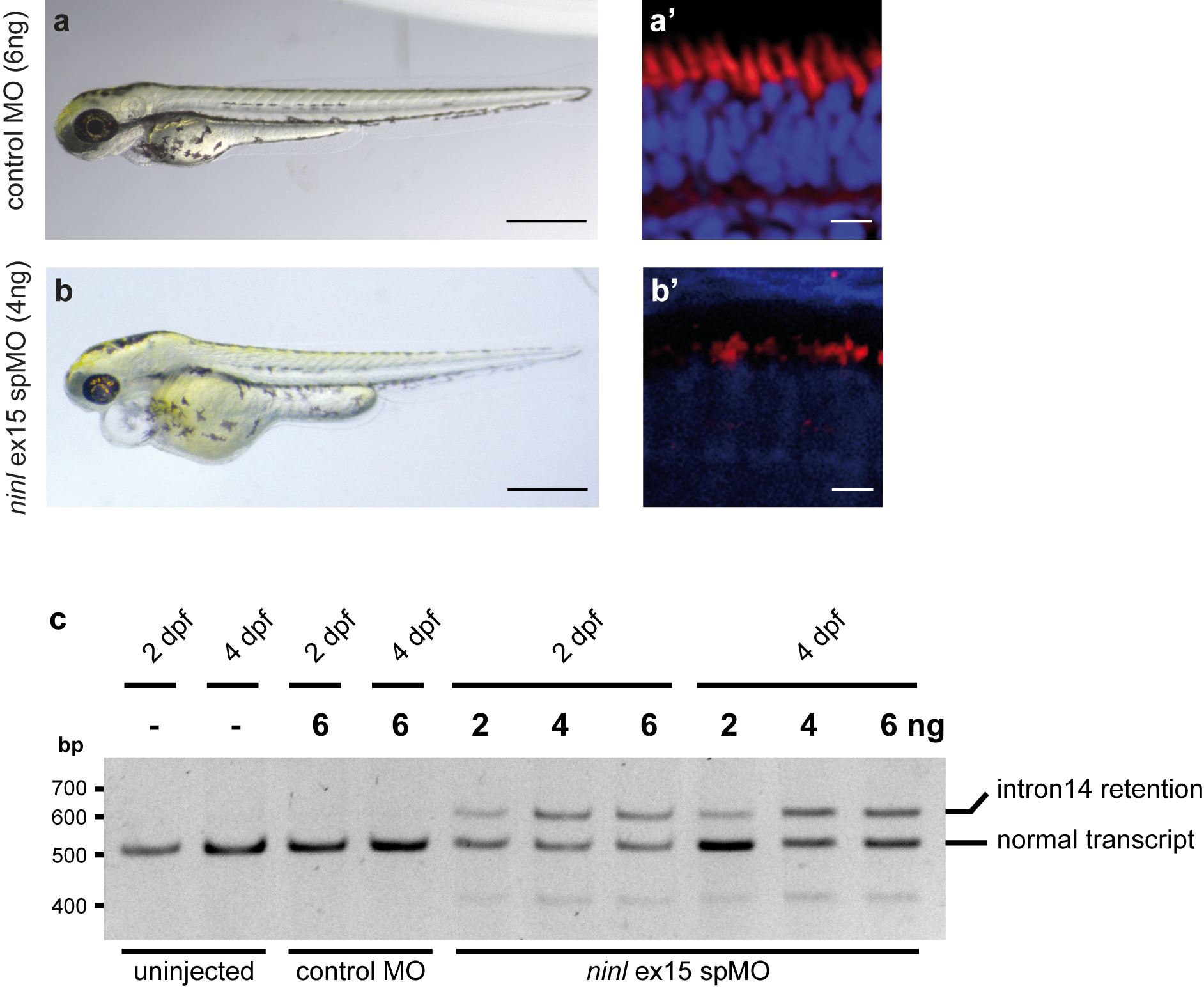

Supplement: S5 Fig — (a-b) Injection of 4ng ninl ex15 spMO (n>100) results in heart edema and small eyes. No defects in body curvature were observed in comparison to control MO-injected larvae. (a’, b’) Analyses of bodipy-stained retinas of ninl ex15 spMO-injected larvae (n = 10) revealed defects in photoreceptor outer segment formation (10 of 10) similar to those observed in ninl atgMO-treated larvae, whereas stained retinas of control MO-injected larvae (n = 10) appeared normal (10 of 10). Scale bars represent 500μm (a-b) and 5μm (a’-b’). (c) RT-PCR analysis on RNA isolated from 25 larvae that were either uninjected, injected with control MO (6ng) or injected with various amounts of ninl ex15 spMO (2, 4, 6ng), collected at two different time points after injection (2 dpf and 4 dpf). One PCR product of the expected length (~500bp) was obtained from RNA from uninjected and control MO-injected larvae. Sequence analysis revealed that this was the predicted transcript including exons 13–16. RT-PCR analysis on RNA obtained from the morphant larvae resulted in two products: Sequence analysis of both fragments revealed that the shorter product is the predicted wild-type transcript (ex13-16) and that the longer transcript in addition includes the entire intron 14 (85 bp), resulting in premature termination of translation already after two codons in intron 14. This aberrant splicing persists at 4dpf. (TIF) [file pgen.1005575.s005.tif]
