# Supplementary material for: The Ciliopathy Protein CC2D2A Associates with NINL and Functions in RAB8-MICAL3-Regulated Vesicle Trafficking
Source: PLoS Genet. 2015 Oct 20;11(10):e1005575. doi: 10.1371/journal.pgen.1005575 (PMC4617701; doi:10.1371/journal.pgen.1005575)
Supplement: S2 Table — (DOCX) [file pgen.1005575.s007.docx]

**S2Table: siRNA sequences**

| **RefSeq Accession Number** | **Gene Symbol** | **Gene ID** | **siRNA ID** | **Sense siRNA Sequence** | **Antisense siRNA Sequence** |
| --- | --- | --- | --- | --- | --- |
| NM_025176 | NINL | 22981 | s22764 | GGAGCAUCGUGUGACCAUUtt | AAUGGUCACACGAUGCUCCtc |
| NM_025176 | NINL | 22981 | s22763 | GCCUGAAUCAGGAACAUCAtt | UGAUGUUCCUGAUUCAGGCat |
| NM_025176 | NINL | 22981 | s22765 | CAGUGAGUAUAGAAACGGAtt | UCCGUUUCUAUACUCACUGga |
| XM_940346 | CC2D2A | 57545 | s33278 | GCCUAUUCCUGAGACUACUtt | AGUAGUCUCAGGAAUAGGCag |
| XM_940346 | CC2D2A | 57545 | s33277 | GAAUCAGUGAUAAAUCGUUtt | AACGAUUUAUCACUGAUUCtc |
| XM_940346 | CC2D2A | 57545 | s33279 | CCAUGAUUCUGCACGAAAAtt | UUUUCGUGCAGAAUCAUGGaa |
