# Supplementary material for: The Ciliopathy Protein CC2D2A Associates with NINL and Functions in RAB8-MICAL3-Regulated Vesicle Trafficking
Source: PLoS Genet. 2015 Oct 20;11(10):e1005575. doi: 10.1371/journal.pgen.1005575 (PMC4617701; doi:10.1371/journal.pgen.1005575)
Supplement: S3 Table — (DOCX) [file pgen.1005575.s008.docx]

**S3 Table: Primer sequences**

| **Name** | **Fw Primer sequence (5'-3')** | **Rv primer sequence (5'-3')** |
| --- | --- | --- |
| NINL#1 | GGAAGGTTTTGTGGCTGTG | AGGCAGCTGATTCCAAAG |
| NINL#2 | CTGGGACAGGAGGCTTCTAC | ATTTGGTCACTCTGCTGCTG |
| CC2D2A#1 | AGGAAGCGTAACACCCAATG | CCTGGACACCTCCTTGTTG |
| CC2D2A#2 | AGGGTCCAACTGCCTATGTG | ACAGCCCACATTTTTCAAGG |
| GUSB | AGAGTGGTGCTGAGGATTGG | CCCTCATGCTCTAGCGTGTC |
